# Comprehensive Quantitative Urinary Steroid Profiling of 29 Steroids Using Liquid Chromatography‐Tandem Mass Spectrometry

**DOI:** 10.1002/ansa.70087

**Published:** 2026-04-30

**Authors:** Joshua T. Bain, Fozia Shaheen, Alessandro Prete, Lorna C. Gilligan, Angela E. Taylor

**Affiliations:** ^1^ Department of Metabolism and Systems Science, College of Medicine and Health University of Birmingham Birmingham UK; ^2^ National Institute for Health and Care Research (NIHR) Birmingham Biomedical Research Centre Birmingham UK

**Keywords:** androgens, glucocorticoids, LC‐MS/MS, mineralocorticoids, steroid precursors, steroid profiling, urine

## Abstract

Steroids are critical for numerous physiological processes; disruption in their metabolism is associated with numerous endocrine disorders. Steroid quantification is essential to improve the understanding and diagnosis of these pathologies. Historically, urinary steroid profiling has been performed using low‐throughput gas chromatography mass spectrometry (GC‐MS), providing holistic coverage of steroid classes with low cross‐reactivity. Here, we translate our previous GC‐MS urinary steroid profile to a liquid chromatography tandem‐MS (LC‐MS/MS) platform, offering a validated, comprehensive overview of steroid metabolism with comparatively low sample preparation times and increased throughput. Urinary steroids were enzymatically hydrolysed and extracted via C_18_ solid‐phase extraction. Quantification was conducted using a triple quadrupole mass spectrometer. Chromatographic separation of 27 analytes was completed in 16 min using a C_18_‐T3 column. Due to chromatographic co‐elution of tetrahydrocortisol and 5α‐tetrahydrocortisol, a second injection was required on a BEH‐C_18_ column for their separation. Lower limit of quantification (LLOQ) ranged from 2 to 20 ng/mL, with accuracy (bias) ranging from ‐18.7% to 19.9%, and precision (percentage coefficient of variation [%CV]) ranging from 4.0% to 18.6%. Matrix effects remained within the ideal range <±15% for all steroids. Recovery ranged from 76% to 103%, and intra‐ and inter‐assay imprecision (CV) ranged from 0.8% to 14.9%. In 40 healthy volunteers, most analytes were detected above the LLOQ in over 95% of samples, although tetrahydro‐aldosterone (85%), 5‐pregenediol (68%), and pregnanetriol‐one (59%) demonstrated lower quantification rates. Diurnal and sex‐based variations were observed, with excretion levels significantly higher during daytime and in males. This robust, high‐throughput LC‐MS/MS method facilitates the simultaneous quantification of multiple steroid classes, enhancing its utility for clinical and research applications in endocrine science.

## 1 Introduction

**FIGURE 1:**
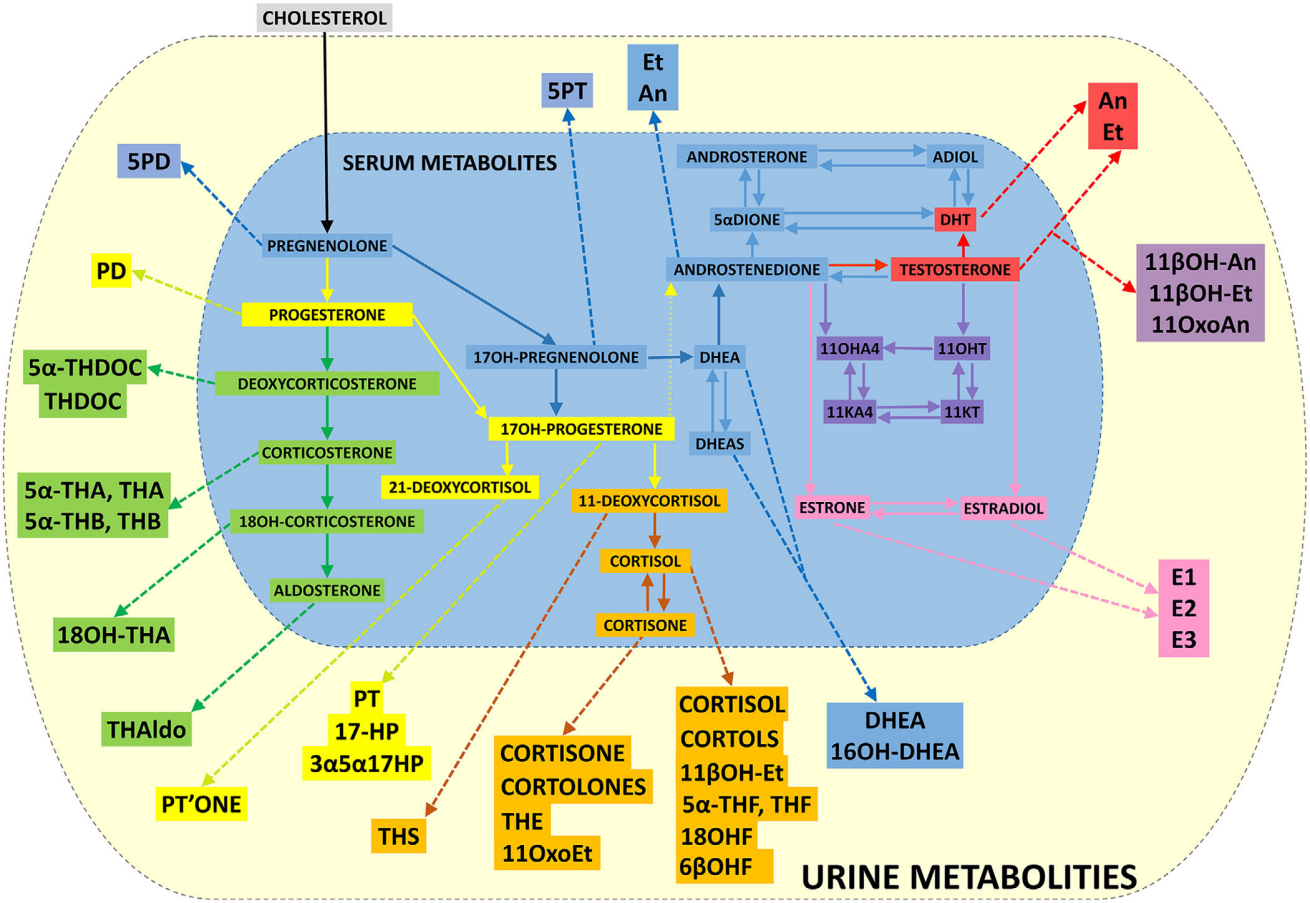
Steroid metabolic pathways showing major steroid metabolites derived from cholesterol, common pathways in their biosynthesis and the relationship between steroids typically found in serum (blue area) versus steroids excreted in urine (yellow area).

Steroid hormones regulate a variety of critical biological processes, including sexual differentiation, stress adaptation, anabolic and catabolic metabolism, and blood pressure. Originating from cholesterol, steroid hormones are biosynthesised via multiple enzymatic steps (Figure 1) in endocrine and other organs, particularly the adrenal cortex and gonads [1]. Post‐synthesis, they undergo hepatic and renal metabolism and are excreted in urine. Analysis of the urinary steroid profile enables characterisation of systemic steroidogenesis.

Dysregulation of steroid pathways leads to various clinical conditions. Excess glucocorticoids define Cushing's syndrome [2], hyperaldosteronism characterises primary aldosteronism [3], adrenal cancer is associated with elevated steroid output [4], and androgen excess is a typical feature of polycystic ovary syndrome [5, 6]. Enzyme deficiencies, such as those seen in congenital adrenal hyperplasia [2], also disrupt steroidogenesis. These pathologies are reflected by unique urinary steroid profiles. To determine which steroids are characteristic of specific diseases/conditions, multi‐steroid quantification from a single sample is advantageous.

Gas chromatography‐mass spectrometry (GC‐MS) has long served as the standard for urinary steroid profile analysis due to its high chromatographic resolution and reproducibility [4, 7, 8]. However, it is limited by laborious derivatisation steps, long run times and therefore high operational costs. These constraints hinder its applicability in high‐throughput, large sample number studies and limit the ability for these methods to be validated to modern standards. Immunoassays are used clinically for the analysis of some steroids but are limited to single‐analyte‐single‐assay detection and significantly hindered by cross‐reactivity, potentially producing false positive results and inaccuracies [9].

Liquid chromatography‐tandem MS (LC‐MS/MS) can be used for urinary steroid profiling (USP), allowing simultaneous quantification of multiple analytes with minimal sample preparation compared to GC‐MS, and compared to immunoassays, LC‐MS/MS provides improved specificity, sensitivity, and accuracy.

Current clinical LC‐MS/MS assays are often focused on small panels of steroids, typically for specific clinical applications, keeping method complexity low. To improve inter‐laboratory standardisation, external quality assessment schemes are available for some, typically serum, steroids [10]. To improve standardisation further, companies such as Chromsystems [11] and Tecan [12] have developed kits with calibrators and internal standards included, though again with a focus on serum steroid profiles. Implementation of large, novel steroid profiles, especially in urine, remains challenging due to additional sample preparation requirements, high costs, complex operation, and limited inter‐laboratory standardisation.

Steroidogenesis is a complex process, and focusing on single analytes or classes of steroids can limit interpretation. Methods that analyse larger panels of steroids are required to provide a holistic view of steroid metabolism, especially important for discovery science, where the steroid(s) of interest may be unknown, or when investigating off‐target effects. Larger panels are methodologically and technically challenging due to increased complexity and pseudo‐optimal conditions, as the method is not fully optimised for any single steroid.

In research laboratories, LC‐MS/MS multi‐steroid profiling has enabled the investigation of several steroid‐related conditions. A key example is our 15 steroid USP method for diagnosing adrenocortical carcinoma (ACC), which was refined from an initial screen of 32 GC‐MS quantified steroids [4] and translated to an LC‐MS/MS assays, focusing on fewer analytes [6, 13].

Previously, discovery research has relied on GC‐MS as a discovery tool to identify diagnostically or clinically relevant steroids, typically investigating only a small number of samples due to the high cost and time demands of the technique. In this study, we translate and validate a previously utilised GC‐MS [4, 7] method for USP to an LC‐MS/MS platform, with the aim of increasing throughput and reducing the cost per sample. To provide a similar specificity to our GC‐MS assay [4, 7], we have attempted to separate 20α‐β and 5α‐β pairs, An/Etio, THB/5αTHB, 11βOHAn/11βOHEt, α‐cortol/β‐cortol, α‐cortolone/β‐cortolone, 11OxoAn/11OxoEt, and 5α‐tetrahydrocortisol (5αTHF) and 5βTHF. This paper establishes and validates an LC‐MS/MS USP method, using our published GC‐MS method as a basis, providing a clinically relevant urinary steroid profile including steroid precursors, mineralocorticoids, glucocorticoids, androgen precursors, androgens and 11oxygenated androgens. Additionally, we were able to demonstrate reproducible, reliable use of dehydrated ions as the parent ion for steroid quantification.

Our validation procedure ensured our method was accurate, precise, reproducible, sensitive, and high‐throughput. This method is designed for discovery science, offering excellent analytical performance with comprehensive coverage across steroid classes, supporting both biomarker discovery and clinical investigation. The extensive validation procedure described here highlights the utility of this method, or components thereof, for clinical translation, providing a foundation for simplified, clinically translatable research targeting smaller condition‐specific steroid panels.

## 2 Materials and Methods

### 2.1 Preparation of Standards and Quality Controls

Twenty‐nine reference standards and fourteen isotopically labelled internal standards were sourced from Merck Life Science (Gillingham, UK), Steraloids (Newport, USA) and IsoSciences (Philadelphia, USA). The suppliers, trivial names, chemical names, and steroid classes for all standards and internal standards are listed in Table S1. Purity and identity were verified using GC‐MS following methyloxime‐trimethylsilyl derivatisation [7]. Standards were dissolved at 1 mg/mL in ultra‐high‐performance liquid chromatography (UHPLC)‐grade methanol (Biosolve, Dieuze, France) and stored at −80°C. All steroid‐containing samples were stored in hexamethyldisilazane‐treated glass vials (Fisher Scientific, Loughborough, UK) to minimise diffusion/sticking of steroids to the glass [14]. Thirteen calibration samples and three quality control (QC) samples were generated by spiking the surrogate matrix across a concentration range (0–3000 ng/mL; extended to 6000 ng/mL for analytes with high urinary excretion, Table S2). The surrogate matrix selected was phosphate‐buffered saline (PBS) with 0.1% bovine serum albumin (BSA), chosen due to the ease of procurement and comparability in terms of extraction efficiency and matrix effects to the steroid‐free synthetic urines, Surine, and Sigmatrix Urine Diluent (Table S3).

### 2.2 Sample Extraction Protocol

Urine samples (200 µL) were spiked with 20 µL of an internal standard mixture and 200 µL enzymatic hydrolysis buffer containing the Helix pomatia enzyme to deconjugate steroids from their glucuronidated and/or sulfated conjugates. The enzymatic hydrolysis buffer consisted of 0.2 mol/L acetic acid, 0.2 mol/L sodium acetate, 17 mmol/L sodium ascorbate and 66 enzyme units/mL of sulfatase from Helix pomatia in deionised water (Milli‐Q purity water system); all reagents were purchased from Merck Life Science (Gillingham, UK). After a 3‐hour incubation at 60°C, samples were extracted using a fixed 96‐well C_18_ solid‐phase extraction (SPE) plate (100 mg) under positive pressure (Biotage, Hengoed, UK). The SPE plate was pre‐conditioned with 800 µL of UHPLC‐grade methanol, then washed with 800 µL of UHPLC‐grade water. The hydrolysed urine samples were applied to a SPE cartridge, which was then washed with a further 800 µL of UHPLC‐grade water, and finally the steroids were eluted with 800 µL of methanol. The methanol eluent was dried under a nitrogen stream at 50°C and reconstituted in 200 µL of 50% (v/v) methanol and water. Additional biological QC samples, consisting of a pooled 24‐h urine from six volunteers, were extracted in triplicate alongside each sample batch.

### 2.3 Chromatography

Chromatography was optimised to include steroids of multiple classes with varying polarity and to avoid co‐elution of steroids with identical masses, while keeping the run time as short as possible, ideally under 20 minutes. Chromatography was performed on a Waters Acquity UHPLC system with a 50 µL loop (Waters Ltd, Wilmslow, UK), using an Acquity UPLC HSS T3 column (1.8 µm, C_18_ 100 Å, 2.1 × 50 mm) (Waters Ltd), maintained at 60°C. A 10 µL volume of the reconstituted sample was injected. Mobile phase A consisted of water, and mobile phase B of methanol, both with 0.1% (v/v) formic acid (95%) (Biosolve, Dieuze, France). To separate analytes, an optimised gradient method was applied with a flow rate of 0.6 mL/min over 18 min, starting at 30% B. A linear gradient was applied over the first minute to 28% B, followed by a second linear gradient over 5 min to 39% B. A third linear gradient was then applied over 7 min to 56% B. Finally, a fourth linear gradient was applied for 3.5 min to 70% mobile phase B. This was followed by a wash step at 98% B (0.7 min) and re‐equilibration at initial conditions (30% B) for 0.8 min prior to the next injection. The autosampler was maintained at 10°C.

Co‐elution of 5αTHF and tetrahydrocortisol (5β‐THF) was present in the analytical run under the above conditions. To achieve the baseline separation of these steroids required for quantification, a second 10 µL injection from the same sample was analysed using an Acquity UPLC BEH C18 column (1.7 µm, C_18_ 130Å, 2.1 × 50 mm) (Waters Ltd) at 60°C. This method used the same mobile phases as described above, again at a flow rate of 0.6 mL/min. Separation was achieved using a one‐minute hold at 30% of mobile phase B, followed by a linear gradient applied for 4 min to 60% B. This was followed by a wash step at 98% B (0.7 min) and re‐equilibration at initial conditions, 30% B for 0.8 min prior to the next injection.

### 2.4 Mass Spectrometry

Mass detection was performed using a Waters Xevo TQ‐XS mass spectrometer equipped with an electrospray ionisation (ESI) source operating in positive ionisation mode (Waters Ltd, UK). The capillary voltage was set to 1.5 kV, with a source temperature of 150°C and a desolvation temperature of 600°C. Cone gas flow was maintained at 1200 L/h. MassLynx 4.2 software (Waters Ltd) was used for instrument control and optimisation of mass transitions. Qualifier and quantifier mass transitions, cone voltages, and collision energies were optimised for each steroid using direct infusion at 20 µL/min of a 500 nM solution prepared in 50% (v/v) methanol/water. Mass transitions were optimised individually for each analyte, and acquisition parameters were configured to collect at least 12 data points per chromatographic peak for accurate quantification. Peak area ratios of quantifier to qualifier ions were continually monitored to evaluate cross‐reactivity from isobaric steroid interferences and matrix effects.

Peak area ratios of analyte to internal standard (response) were plotted against the theoretical concentrations of the calibrators. A linear least squares regression with 1/*x* weighting was applied to generate the standard curves. Retention times, quantifier and qualifier mass transitions, collision energies, and cone voltages for all target analytes and internal standards were fully optimised. Peak integration and quantification were performed using MassLynx 4.2 software, and post‐analysis data manipulation was performed in Microsoft Excel.

### 2.5 Validation

Validation was performed following protocols from published methods and established guidelines as a framework [15]. For all steroids validation was conducted using both spiked surrogate matrix and biological samples.

#### 2.5.1 Recovery and Matrix Effects

Recovery and matrix effects were determined for 24‐h urine collections pooled from both male and female donors, as well as individual male and female samples. Aliquots of each sample set were extracted in triplicate under three conditions: (1) without spiking (endogenous); (2) spiked with known concentrations of all steroids before extraction (pre‐extraction); and (3) spiked with all steroids after extraction (post‐extraction). These samples were compared to assess percentage recovery using:


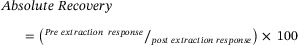




Additionally, the reconstitution solvent (50% UHPLC‐grade methanol in Milli‐Q water, v/v) was spiked with all target steroids at known concentrations to serve as the non‐extract control (non‐extraction response). Matrix effects were evaluated at three concentrations (50, 100, and 200 ng/mL) by comparing the endogenous and post‐extraction spike samples, employing the following equation:


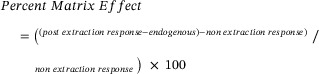




Matrix effects were determined after normalisation to the internal standard in order to determine the impact of the sample matrix on assay performance. Mean values between −15% and +15% were considered acceptable.

#### 2.5.2 Limits of Quantification

The lower limit of quantification (LLOQ) was determined as the lowest concentration where 10 sample replicates of spiked surrogate matrix could be measured with an intra‐assay (within‐run) coefficient of variation (CV) ≤20%, and accuracy (bias) calculated from 10 individually prepared samples within ±20% of the theoretical concentration. LLOQ was calculated as ng/mL, and an estimated 24‐hour LLOQ was determined based on an average 24‐hour urine volume of 1400 mL.

The upper LOQ (ULOQ) was determined as the highest concentration where 10 sample replicates of spiked surrogate matrix could be measured with an intra‐assay (within‐run) CV ≤15%, and accuracy (bias) calculated from 10 individually prepared samples within ±15% of the theoretical concentration.

#### 2.5.3 Linearity

For each steroid, the ratio of analyte peak area to internal standard peak area was plotted against the theoretical concentrations. To be deemed an acceptable calibration series, each data point above the LLOQ and below the ULOQ should have an accuracy (bias) not exceeding ±15% compared to the theoretical concentration. Additionally, the regression model should give an R^2^ value greater than 0.99, indicating a strong linear relationship between concentration and response.

#### 2.5.4 Imprecision

In addition to the LLOQ and ULOQ, intra‐assay (within‐run) imprecision was assessed by spiking the surrogate matrix at three concentrations: 20, 300, and 800 ng/mL for most analytes (40, 600, and 1600 ng/mL for all analytes with higher expected excretion, An, Et, 5αTHF, THF, and THE). These concentrations were chosen as they represent concentrations of low, medium, and highly excreted steroids in urine, which range from <20 to >5000 µg/24‐h. Thus, providing a relevant comparator, this is particularly important where parameters stated in the guidelines, such as 3 × LLOQ, were not biologically relevant. Additionally, a male 24‐h urine sample, a female 24‐h urine sample, and a pooled urine sample from six donors were extracted. Each sample type was extracted and analysed 10 times in the same batch to assess intra‐assay (within‐run) imprecision. A percentage deviation (variance CV%) in the calculated concentrations across all 10 extracts of ≤15% was considered acceptable.

Inter‐assay (between‐runs) imprecision was evaluated by analysing male and female and pooled 24‐h urine samples across three independent analytical runs on different days. Each sample type was extracted 10 times per analytical batch. The CV% was calculated across all runs; a CV% ≤15% was considered acceptable.

#### 2.5.5 Accuracy

In addition to the LLOQ and ULOQ, 10 individually prepared samples of surrogate matrix were spiked with all analytes at three different concentrations representative of excretion observed in our participant cohort, 20, 300 and 800 ng/mL or 40, 600 and 1600 ng/mL for An, Et, 5αTHF, THF and THE. A bias from the theoretical concentration within ±15% was considered acceptable (±20% at LLOQ).

#### 2.5.6 Carryover

Carryover was assessed by analysing a blank sample (50/50 methanol/water) immediately following the injection of the highest calibration standard (3000 ng/mL for most steroids or 6000 ng/mL for An, Et, 5αTHF, THF, and THE). The percentage carryover was calculated by comparing the peak area of the analyte in the blank sample to the peak area in the concentrated sample. A carryover of less than 2% was deemed acceptable.

#### 2.5.7 Stability

To assess the stability of analytes after freeze/thaw cycles, three aliquots of pooled 24‐h urine were analysed after 3 freeze/thaw cycles. To assess stability of post‐preparative samples, 10 aliquots of pooled 24‐h urine were run, the plate was then re‐sealed and stored at ‐20°C for 2 months, after which it was re‐analysed. Long‐term stability of stored samples was evaluated by analysis of multiple stored aliquots of a pooled 24‐h urine sample across 10 analytical batches from January 2024 to June 2025 (18 months). For all stability assessments, quantitation was compared, and the variance CV% was calculated for all steroids; a CV% ≤15% was considered acceptable.

### 2.6 Clinical Samples

To assess the utility of this method in clinical samples, urinary excretion of steroids was quantified in 40 healthy volunteers (20 females aged 24–69 years and 20 males aged 22–74 years). Exclusion criteria included pregnancy and use of steroids such as oral contraceptives. All clinical samples were obtained from patients who provided full informed, written consent prior to recruitment. Samples left at room temperature for more than 3 days post‐collection were discarded to avoid bacterial growth, which may alter steroid quantification. Participants collected separate day and night urine samples, from which 24‐h steroid excretion was calculated. The use of day, night and 24‐h collections reflects typical clinical urine collection protocols. During sample collection, volunteers were asked to discard their first morning void urine, before starting day collection and continue until the participant was preparing for bed, at which time a final urine was collected into the day bottle, and the number of hours of collection was recorded. The night sample collection was then started, which continued until 24 hours from the start of the day collection, including the morning void. When collecting urine samples, an aliquot should be removed from the well‐mixed sample, centrifuged and stored at −20°C as soon as possible. Steroid excretion was calculated in ng/mL and normalised to µg/sample by multiplying by the sample volume (for day or night collections). 24‐h collections were calculated by the same means and are reported in µg/24 h.

## 3 Results and Discussion

The method meets acceptable limits for sensitivity, specificity, linearity, accuracy, precision, matrix effects, recovery, and carryover across the concentration range observed within the human urinary steroid metabolome (∼10–5000 µg/24 h). The inclusion of 29 steroids across key functional classes: precursor, steroids, mineralocorticoids, glucocorticoids, and androgens provides broad metabolic coverage perfect for discovery investigations. The simultaneous quantification of multiple steroids provides an integrated view of steroid biosynthesis and metabolism, facilitating exploratory endocrine research.

### 3.1 Mass Spectrometry and Chromatography

Each steroid was independently infused into the mass spectrometer and analysed in both positive and negative ionisation modes. Where intact protonated molecular ions were observed, these were introduced into the collision cell and collision energy was increased until three stable product ion transitions were identified. However, for several steroids, intact molecular ions were not detectable, and ionisation was dominated by dehydration products, resulting in [M‐H_2_O]+ or [M‐2H_2_O]+ parent ions. Attempts to suppress water loss through source optimisation were unsuccessful, therefore source conditions were optimised to reproducibly generate stable water‐loss ions (desolvation temperature 600°C, source temperature 150°C, nitrogen desolvation gas flow 1200 L/hr). Under these optimised conditions, water‐loss transitions were highly reproducible and therefore suitable for full method validation, enabling inclusion of steroids that would otherwise be excluded due to no protonated molecular ion formation. All analytes formed either [M+H]+, [M‐H_2_O]+ or [M‐2H_2_O]+ ions (Table 1). The reproducible use of water‐loss ions was confirmed across two different triple quadrupole platforms (Xevo TQ‐MS and Xevo XS), despite moderate differences in source design, demonstrating the robustness of the approach. Nonetheless, transfer of the assay to other vendor platforms may require substantial source re‐optimisation, particularly where source geometry or desolvation efficiency differs.

**TABLE 1:** Nomenclature, retention times, molecular weight, ionised species, quantifier and qualifier mass transitions, cone voltages and collision energies of target steroids and internal standards.

Analyte (Trivial name)	Retention time (min)	Mol weight and mol ion species	Mass transition (m/z) quantifier (qualifier)	Cone voltage (V) quantifier (qualifier)	Collision energy (eV) quantifier (qualifier)
An (Androsterone)	13.6	290.44 [M+H]^+^	291.1 > 273.1 (291.1 > 255.1)	18 (18)	14 (8)
Et (Etiocholanolone)	13.2	290.44 [M+H]^+^	291.1 > 255.1 (291.1 > 273.1)	12 (12)	14 (8)
11βOHAn (11‐hydroxy‐androsterone)	8.4	306.44 [M+H]^+^	307.1 > 289.2 (307.1 > 271.1)	12 (12)	14 (12)
DHEA (Dehydroepi‐androsterone)	10.4	288.42 [M+H]^+^	289.0 > 253.1 (289.0 > 271.1)	12 (12)	10 (8)
5PT (5‐Pregenetriol)	10.3	334.49 [M‐2H_2_O]^+^	299.1 >281.1 (299.1 > 157.0)	16 (16)	12 (18)
5PD (5‐Pregenediol)	13.6	318.49 [M‐2H_2_O]^+^	301.1 > 283.1 (283.1 > 105.0)	24 (14)	20 (22)
THAs (Tetrahydro‐dehydro‐corticosterone)	9.1	348.48 [M+H]^+^	349.1 > 331.1 (331.1 > 104.9)	28 (46)	10 (42)
THB (Tetrahydro‐corticosterone)	8.6	350.49 [M‐2H_2_O]^+^	315.1 > 297.1 (333.1 > 315.1)	36 (36)	10 (10)
5αTHB (5α‐Tetrahydro‐corticosterone)	8.8	350.49 [M‐2H_2_O]^+^	315.1 > 279.1 (315.1 > 297.1)	32 (32)	10 (12)
THAldo (Tetrahydro‐aldosterone)	4.9	364.5 [M‐H_2_O]^+^	347.1 > 329.1 (347.1 > 391.1)	6 (6)	10 (14)
THDOC (Tetrahydro‐deoxycorticosterone)	13.2	334.49 [M‐H_2_O]^+^	317.1 > 299.1 (317.1 > 281.1)	22 (22) `	10 (10)
PD (Pregnanediol)	15.8	320.51 [M‐H_2_O]^+^	285.1 > 189.1 (285.3 > 92.6)	14 (22)	20 (30)
17HP (17‐hydroxy pregnanolone)	14.7	334.49 [M‐2H_2_O]^+^	299.1 > 105.0 (335.1 > 317.1)	22 (22)	36 (14)
PT (Pregnanetriol)	14.9	336.51 [M‐2H_2_O]^+^	301.1 > 90.9 (301.1 > 283.1)	20 (20)	18 (24)
PTONE (Pregnanetriol‐one)	9.7	350.49 [M‐H_2_O]^+^	333.1 > 315.1 (314.1 > 90.9)	16 (16)	16 (10)
THS (Tetrahydro‐11‐deoxycortisol)	12.3	350.49 [M+H]^+^	351.1 > 315.1 (351.2 > 90.9)	12 (12)	14 (10)
F (Cortisol)	5.0	362.46 [M+H]^+^	363.1 > 121.0 (363.1 > 90.9)	24 (24)	28 (16)
β‐cortol	7.5	368.52 [M‐H_2_O]^+^	333.2 > 273.2 (367.2 > 331.2)	24 (24)	16 (22)
α‐cortol	6.6	368.52 [M‐H_2_O]^+^	331.2 > 273.2 (367.2 > 331.2)	24 (24)	16 (22)
11βOHEt (11‐Hydroxy etiocholanolone)	8.1	306.44 [M+H]^+^	307.2 > 271.1 (307.2 > 253.2)	12 (12)	14 (12)
18OHF (18‐Hydroxy cortisol)	2.8	378.46 [M+H]^+^	379.2 > 267.2 (383.2 > 271.2)	32 (32)	18 (28)
E (Cortisone)	4.6	360.44 [M+H]^+^	361.0 > 163.0 (361.0 > 104.9)	32 (32)	26 (38)
THE (Tetrahydrocortisone)	8.2	364.48 [M+H]^+^	365.2 > 347.2 (365.2 > 329.2)	12 (12)	10 (14)
α‐cortolone	7.6	366.49 [M‐2H_2_O]^+^	331.1 > 271.2 (331.1 > 253.2)	4 (4)	18 (22)
β‐cortolone	8.2	366.49 [M‐2H_2_O]^+^	349.2 > 311.2 (370.2 > 352.2)	28 (28)	20 (22)
11OxoAn (11‐Oxo‐androsterone)	8.6	304.42 [M+H]^+^	305.1 > 269.1 (305.1 > 147.1)	26 (26)	10 (12)
11OxoEt (11‐Oxo‐etiocholanolone)	8.4	304.42 [M‐H_2_O]^+^	287.0 > 229.2 (287.0 > 269.2)	4 (4)	18 (18)
THF (Tetrahydrocortisol)	3.6	366.49 [M‐H_2_O]^+^	349.1 > 301.1 (336.2 > 300.2)	28 (2)	8 (12)
5αTHF (5α‐Tetrahydrocortisol)	3.5	366.49 [M‐2H_2_O]^+^	331.1 > 313.1 (331.1 > 295.1)	26 (10)	8 (10)

Internal standards					
Internal standard (Trivial name)	Retention time (min)	Mol weight and mol ion species	Mass transition (*m/z*) quantifier qualifier	Cone voltage (V) quantifier (qualifier)	Collision energy (eV) quantifier (qualifier)
An‐d4 (Androsterone‐d4)	13.5	294.44 [M+H]^+^	295.1 > 277.2 (295.1 > 260.2)	12 (12)	8 (16)
Et‐d5 (Etiocholanolone‐d5)	13.1	295.44 [M+H]^+^	296.2 > 260.2 (296.2 > 220.1)	12 (12)	18 (20)
DHEA‐d6 (Dehydroepi‐androsterone‐d6)	10.3	294.42 [M+H]^+^	295.1 > 277.1 (295.1 > 258.4)	12 (22)	10 (14)
18OHF‐d4 (18‐Hydoxy cortisol‐d4)	2.8	382.46 [M+H]^+^	383.2 > 271.2 (383.2 > 289.2)	32 (32)	18 (28)
THAldo‐d6 (Tetrahydro‐aldosterone‐d6)	4.8	370.5 [M‐H_2_O]^+^	353.3 > 316.7 (353.3 > 334.7)	6 (6)	10 (14)
PD‐d5 (Pregnanediol‐d5)	15.7	325.51 [M‐H_2_O]^+^	308.2 > 140.1 (308.2 > 135.1)	14 (14)	20 (20)
PT‐d5 (Pregnanetriol‐d5)	14.9	341.51 [M‐2H_2_O]^+^	306.2 > 140.1 (306.2 > 135.1)	20 (20)	24 (18)
THS‐d5 (Tetrahydro‐11‐deoxycortisol‐d5)	12.3	355.49 [M+H]^+^	356.2 > 320.2 (356.2 > 302.1)	18 (18)	12 (12)
F‐d4 (Cortisol‐d4)	4.9	366.46 [M+H]^+^	367.2 > 331.1 (267.2 > 121.1)	24 (24)	24 (16)
THB‐d5 (Tetrahydro‐corticosterone‐d5)	8.6	357.49 [M‐2H_2_O]^+^	320.1 > 302.2 (320.1 > 284.2)	26 (22)	12 (10)
THE‐d5 (Tetrahydrocortisone‐d5)	8.2	369.48 [M+H]^+^	370.2 > 352.2 (370.2 > 334.2)	12 (12)	10 (14)
E‐d7 (Cortisone‐d7)	4.6	367.44 [M+H]^+^	369.1 > 169.0 (369.0 > 121.0)	40 (46)	24 (28)
11OxoEt‐d5 (11‐Oxo‐etiocholanolone‐d5)	8.3	309.42 [M‐H]^+^	310.1 > 292.1 (292.0 > 274.1)	4 (16)	18 (10)
THF‐d5 (Tetrahydrocortisol‐d5)	3.5	371.49 [M‐H_2_O]^+^	336.2 > 300.2 (336.3 > 295.2)	18 (18)	12 (12)

An alternative approach to improve sensitivity could be derivatisation [16]. However, derivatisation introduces experimental variation and would require additional validation experiments regarding derivatisation efficiency and reproducibility, adding significant cost and complexity to the method. Alternatively, alteration of the mobile phase to form adducts such as lithium adducts [17, 18] has been shown to improve sensitivity for some urinary steroids. Wang and colleagues reported this with a 188‐fold increase in THF and a 29‐fold increase in THE sensitivity; this would saturate the detector in our assay. Conversely, some steroids show reduced signal when forming a lithium adduct, for example, androsterone, raising its LLOQ [17]. These shifts in sensitivity would substantially alter our assay and require a full re‐validation.

Mass transition selection was performed using the automated Waters *IntelliStart* software, which identified the three most abundant product ions for each parent ion. The transition with the highest peak area was designated as the quantifier, and the second most intense as the qualifier. Each steroid was injected 100 times to assess transition stability and reproducibility, resulting in two stable transitions per analyte, with the most intense transition used for quantification (Table 1). Quantifier‐to‐qualifier ion ratios and internal standard peak areas were continuously monitored to ensure assay specificity, with no interferences detected in the analysed samples (Tables S4 and S5). This extensive transition stability assessment and internal standard monitoring exceed that reported in several published assays that use fewer internal standards or less stringent validation criteria [19, 20].

**FIGURE 2:**
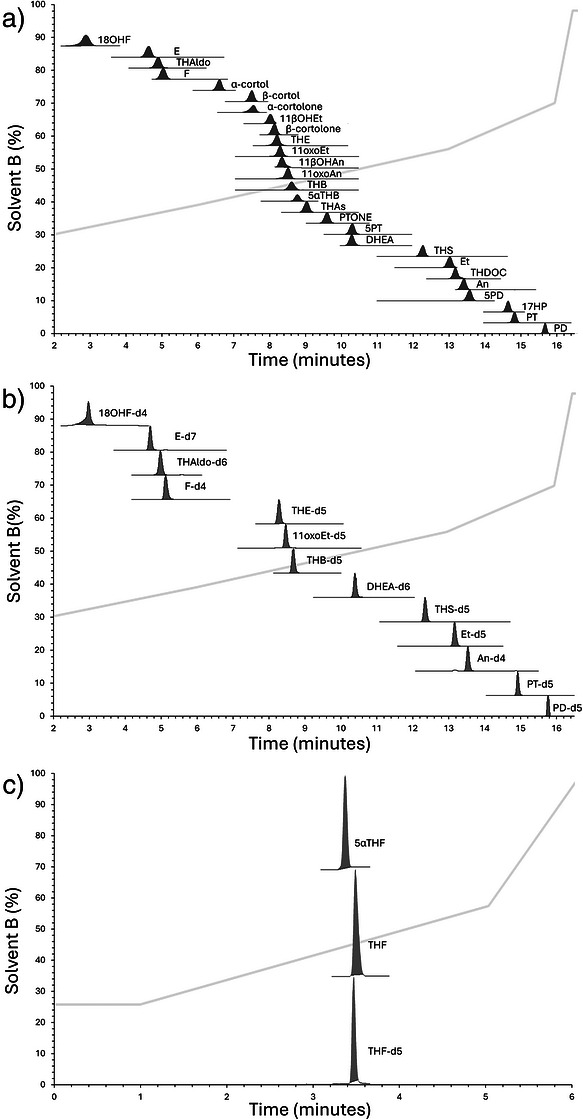
(a) Chromatographic separation of 27 steroids, projected on top of the gradient profile %B (methanol 0.1% formic acid) against time (minutes) HSS‐T3 column. (b) Chromatographic separation of 13 steroids internal standards, projected on top of the gradient profile %B (methanol 0.1% formic acid) against time (minutes), HSS‐T3 column. (c) Chromatographic separation of the second injection to separate THF, 5αTHF and THF‐d5 projected on top of the gradient profile %B (methanol 0.1% formic acid) against time (minutes), BEH column. Both on an Acquity UPLC with Waters Xevo‐XS.

Chromatographic separation was achieved for 27 analytes in a single injection using a methanol/water gradient, followed by wash and equilibration steps, resulting in a total run time of 18 min injection‐to‐injection (Figures 2a,b). Co‐eluting THA and 5αTHA were quantified as total THAs. The reliance on parent ions formed via water loss introduced additional specificity considerations, particularly for steroids sharing identical molecular weights, including those indistinguishable after source‐induced dehydration. These challenges were addressed through chromatographic optimisation to ensure that analytes with the same molecular mass or protoned molecular masses realted to the single or double water losses did not co‐elute. No specificity issues were observed for any steroids included in the panel (Figure 2a,b; Table 1), demonstrating that chromatographic resolution can effectively compensate for mass‐related specificity problems. There are some similar urinary steroid methods in the literature. Hyuck et al. [19] quantified a large number of analytes, overlapping with the present panel, but limited to 10 steroids and internal standard coverage was restricted. A 2017 method by Hines et al. [20] profiled 26 steroids using a high‐resolution quadrupole Orbitrap system, twenty‐four of which were included here; however, this method was not validated to the same extensive criteria, and they only employed four internal standards.

A C_18_‐T3 (tri‐functional C18) column provided sufficient resolving power to separate the steroids, including several biologically critical α/β isomer pairs (An/Etio, THB/5αTHB, 11βOHAn/11βOHEt, α‐cortol/β‐cortol, α‐cortolone/β‐cortolone, and 11OxoAn/11OxoEt). Methods described by Wang et al. [17] and Pan et al. [18] quantified a comparable number of steroids but showed limited overlap with the present assay and did not resolve key 5α/β pairs. In our method, a second injection using a BEH‐C_18_ (bridged ethylene hybrid) column was required to achieve baseline separation of THF and 5αTHF (Figure 2c), ensuring accurate quantification of these major glucocorticoid metabolites.

We included fourteen internal standards spanning the chromatographic range (including first and last eluting steroids 18OHF and PD) to account for matrix effects and ionisation variability. For analytes without matched internal standards, surrogates were chosen based on extraction, structural and/or chromatographic similarities. Further internal standards were not available at the time of this assay development; however, it is probable that performance could be improved with the inclusion of more isotopically matched internal standards, especially for analytes with borderline accuracy or precision. The steroids included here do not represent all urinary steroids but include those most highly excreted and/or important analytes in each class. There is a chance that steroids not included in this assay could cause specificity issues if they co‐elute; however, our matrix effects experiments demonstrated this to be minimal in healthy volunteers.

This method demonstrated reproducible use of water‐loss ions, combined with targeted chromatographic separation, enabling comprehensive, specific USP without derivatisation or adduct formation, while maintaining analytical robustness across a comprehensive steroid panel. In comparison with previously published methods, the present assay offers a unique combination of analyte coverage, chromatographic resolution of stereoisomers, and extensive validation.

### 3.2 Method Validation Performance

Most analytes showed acceptable analytical performance, though 5‐pregenediol (5PD) was problematic, with accuracy outside acceptable criteria at two concentration levels (19.1% and −18.8%). Additionally, a subset of analytes: tetrahydro‐aldosterone (THAldo), 5PD, and pregnanetriol‐one (PTONE) were not quantifiable in ∼15% of samples, reflecting their low excretion levels. This was especially challenging when the 24‐h urine volume was larger than 3000 mL, due to dilution of the steroids.

#### 3.2.1 Calibration Matrix

The selection of an appropriate surrogate matrix for urine for preparation of the calibration series, determination of LLOQ, ULOQ, and accuracy and imprecision assessment poses some issues. Use of a matrix‐matched calibration surrogate would be preferred, but no suitable product was available. Use of charcoal‐stripped urine introduces variability due to donor biology and charcoal activity, leading to significant variation [21, 22]. Therefore, we selected a matrix without steroid interferences, PBS (0.1% BSA). Recovery, extraction efficiency and matrix effects of our surrogate matrix were comparable to other commercially available steroid‐free synthetic urines, Surine, and Sigmatrix Urine Diluent. Use of any of these surrogate matrices, including ours, may lead to over‐ or under‐estimation of steroid concentrations at the limits of quantification. For most steroids, this is not problematic as the LLOQ is well below the expected excretion concentrations; however, caution should be taken when interpreting low excreted steroids less than <20 µg/24 h (DHEA, 5PD, THAldo, PTONE and 11‐oxoAn), especially if the 24‐h collection volume is large (>2500 mL). However, our matrix‐effects experiments with spiked participant urine showed minimal bias between our chosen surrogate and biological samples.

#### 3.2.2 Lower limits of Quantification (LLOQ) and Linearity

LLOQs ranged from 2 to 20 ng/mL (approximately 2.8–28 µg/24 h). At the LLOQ, accuracy (bias) ranged from −18.7.% to 19.9%, and precision (CV) ranged from 4.0% to 18.6%. ULOQs were 3000 ng/mL for most analytes, with higher ULOQs of 6000 ng/mL for An, Et, 5αTHF, THF and THE. At the ULOQ, accuracy ranged from ‐14.3% to 14.5% and precision from 0.9% to 9.5% (Table S6).

All calibration curves were linear across the validated range, with individual calibration points within ±15% of theoretical concentrations and coefficients of determination (*R* ^2^) ≥0.99 for all analytes.

#### 3.2.3 Recovery and Matrix Effects

Recovery and matrix effects were evaluated at three concentration levels. Mean recovery across all analytes was 88% (range 76%–103%). Matrix effects ranged from ‐15.0% to 14.9%, within the predefined acceptability criterion of ±15% for all analytes and concentrations (Tables S6 and S7).

#### 3.2.4 Imprecision, Accuracy and Carryover

Imprecision was assessed using spiked surrogate matrix samples at the LLOQ, ULOQ and low, medium and high expected excretion concentrations (20, 300 and 800 ng/mL or 40, 600 and 1600 ng/mL). Intra‐assay imprecision across all analytes at low‐high concentrations was within acceptable limits, with CVs ranging from 3.4% to 14.1% (Table S6).

Inter‐ and intra‐assay imprecision were further assessed using male, female, and pooled urine samples. Intra‐assay CVs ranged from 0.8% to 12.6%, and inter‐assay CVs ranged from 2.1% to 14.9% for all analytes (Table S8).

Accuracy (bias) was evaluated at low, medium, and high expected excretion concentrations (20, 300 and 800 or 40, 600 and 1600 ng/mL), ranged from ‐14.9% to 14.9% for all analytes at all concentrations, except for 5PD at 20 ng/mL (19.1%) and 300 ng/mL (‐18.8%) (Table S6).

Carryover was ≤0.8% for all analytes except PD, which showed 1.9% carryover. All values met the acceptance criterion of <2% (Table S6).

#### 3.2.5 Stability

Freeze–thaw stability testing demonstrated no significant changes in steroid quantification after three cycles, with CVs ranging from 1.9% to 11.8%. Post‐preparative stability testing showed no differences in quantification when extracted samples were re‐analysed 2 months after initial extraction (CVs 3.2%–14.8%). Long‐term stability studies confirmed that urine samples stored at −20°C for 18 months remained stable, with CVs between 9.6% and 14.8% (Table S9).

### 3.3 Clinical Utility and Context

**FIGURE 3:**
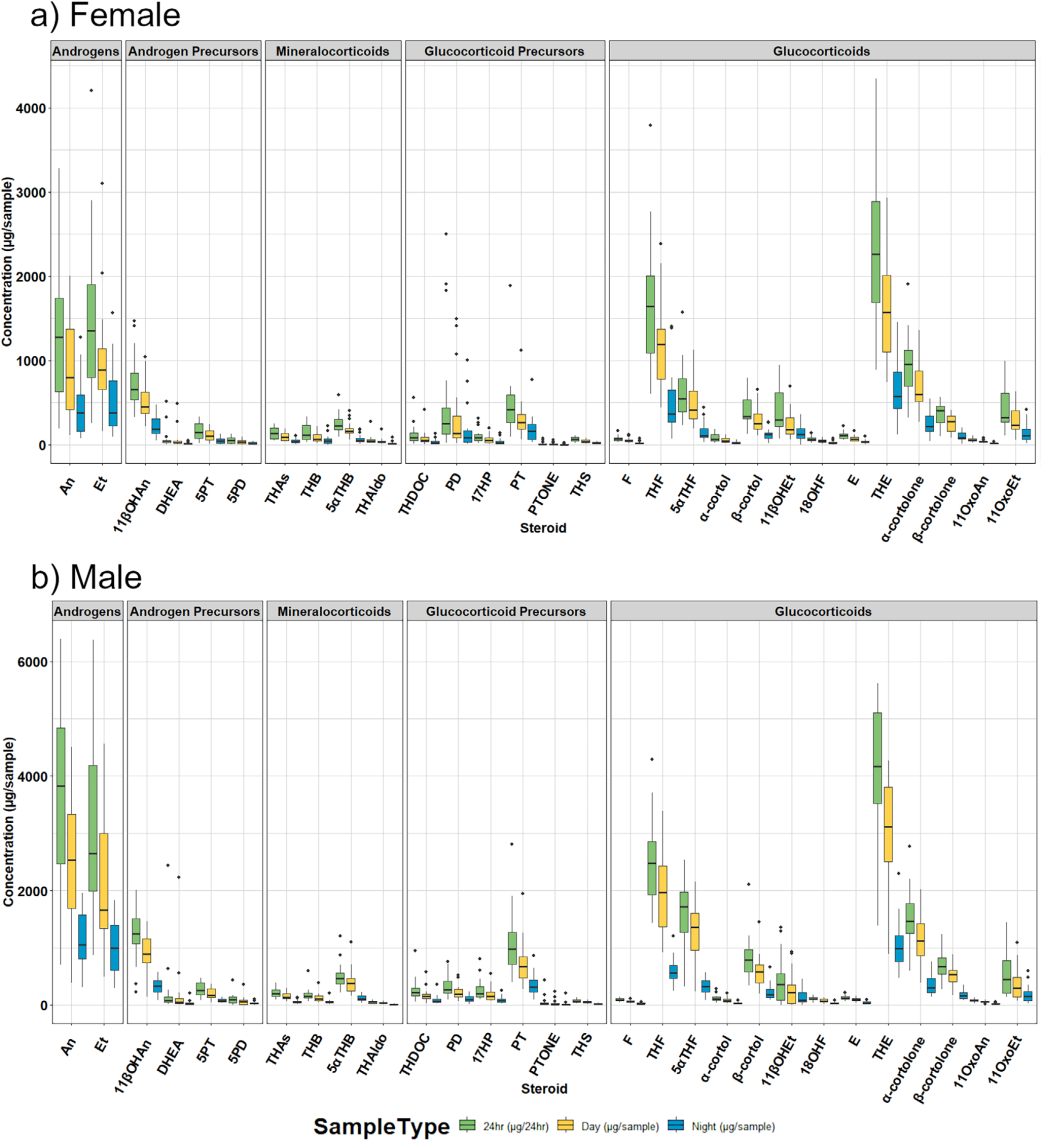
(a) Box and Whisker plot depicting urinary steroid excretion measured within a female population (*n* = 20) 24 h (green, µg/24 h), day (yellow, µg/sample) and night (blue, µg/sample). (b) Box and Whisker plot depicting urinary steroid excretion measured within a healthy male population (*n* = 20) 24 h (green, µg/24 h), day (yellow, µg/sample) and night (blue, µg/sample).

To demonstrate clinical utility, urinary steroid profiles were measured in 40 healthy volunteers. Steroid excretion was calculated in ng/mL and normalised to µg/sample by multiplying by the sample volume (for day or night collections). 24‐h collections were calculated by the same means and are reported in µg/24 h. Concentration ranges are presented in µg/sample and µg/24 h (Table S10; Figure 3) and in nmol/L (Table S11; Figures S1 and S2). Quantification was achieved for most steroids in over 95% of samples. Lower detection frequencies were observed for THAldo (85%), 5PD (68%) and PTONE (59%), consistent with their low endogenous concentrations and poorer ionisability. The analysis of urine from these volunteers was included to show the utility of the method across age and gender, and not to represent a reference range.

Steroid excretion patterns reflected known physiological differences. Excretion was consistently higher during daytime collections than at night and higher in males than in females. For example, median 24‐h THE excretion was 4161 µg/24 h in males compared with 2260 µg/24 h in females, while median 5αTHF excretion was 1718 µg/24 h in males and 545 µg/24 h in females. This aligns with previous reference ranges generated by us using GC‐MS [4] and aligns with established sex differences and diurnal rhythms in steroidogenesis and underscores the importance of sex, age, and time‐specific considerations in clinical research. While the current method is not intended for use in routine clinical biochemistry due to its complexity and data analysis requirements, this assay is ideally positioned for discovery‐phase research, biomarker identification, and mechanistic studies into endocrine or steroid‐related diseases.

This assay could form the foundation for simplified, clinically translatable tests targeting condition‐specific steroid panels. These findings support the utility of this assay for use in a wide age range in both males and females, highlighting the importance of context‐specific sampling in clinical research and diagnostics. The detection of all steroids in most samples highlights the method's comprehensive nature, though the lower quantifiability of DHEA, α‐cortol, THAldo, 5PD, and PTONE in some samples points to the inherent variability in steroid excretion and the need for personalised approaches. If these steroids were primary research targets, samples should be concentrated further, and mass spectrometry parameters optimised.

### 3.4 Methodological Context and Comparison

The transfer of this assay from GC‐MS to LC‐MS/MS offers reduced analysis time, lower per‐sample cost, and improved throughput, while maintaining robust analytical performance. Whilst the use of GC‐MS as a discovery tool in untargeted investigations cannot be overlooked [7, 8] a key strength of this method is its compatibility with high‐throughput analysis without the need for derivatisation, a significant limitation of traditional GC‐MS methods. Unlike GC‐MS, which requires time‐consuming chemical modification of analytes and complex sample handling, LC‐MS/MS offers a streamlined workflow with reduced sample preparation time and increased throughput [6, 13, 23].

Compared with previously published methods, this assay offers a unique combination of analyte coverage, validation depth, and internal standard inclusion. While other LC‐MS/MS approaches have quantified similar numbers of steroids, overlap with the present panel is limited, and several methods employ fewer internal standards or do not distinguish key 5α/β isomer pairs [17, 18, 19, 20]. Methods using lithium adduct formation achieve enhanced sensitivity for some analytes but alter relative signal intensities in a way that would disrupt the balance of the present assay and necessitate complete re‐validation [17, 18].

The analysis of urine, a non‐invasive biofluid available in large volumes, minimises matrix effects relative to serum or plasma and enables 24‐h collections that normalise diurnal variation, particularly important for glucocorticoids. This method provides a foundation for the future development of simplified, clinically targeted steroid panels and will be applied in studies of the menstrual cycle, menopause, endocrine disease, and drug metabolism.

To demonstrate the potential for translating the steroids included in this assay to address specific clinical questions, the method was validated according to various parameters outlined in industry guidelines [15]. As this is a multi‐analyte LC‐MS/MS method, the approach involved compromise as parameters optimised for one steroid may not be ideal for another; thus, a pareto‐optimal solution was achieved to ensure method‐wide reliability.

While we have shown here that LC‐MS/MS improves throughput and cost efficiency, GC‐MS remains valuable for urine steroid profiling due to its superior chromatographic resolution, extensive spectral libraries, and therefore reliability as a reference method for identifying unknown metabolites, as well as coverage of a wide dynamic concentration range. Indeed, these two technologies should be used in a complementary manner, with GC‐MS providing comprehensive reference data and LC‐MS/MS enabling efficient translation into clinical and high‐throughput research.

## 4 Conclusion

We have developed an LC‐MS/MS assay which translates our historic GC‐MS‐based urinary steroid profile. This new method retains the broad range of steroid analyte classes from our GC‐MS method while delivering simplified workflows and reduced sample preparation time, supporting high‐throughput research with wide applications. The method achieves acceptable validation performance for 24‐h, day or night urine collections, enabling accurate, precise steroid quantification. The method retains separation of α/β stereoisomer pairs and demonstrates the applicability and reproducibility of forced water loss transitions in the mass spectrometer source. This simplified workflow can be implemented in endocrine research, diagnostics and therapeutic monitoring.

## 5 Declaration of Use of Artificial Intelligence‐assisted Technologies

Not used.

## Author Contributions

**Joshua T. Bain**: data curation, formal analysis, investigation, methodology, validation, visualisation, writing – original draft and writing – review & editing. **Fozia Shaheen**: data curation, formal analysis, investigation, methodology, supervision, validation, visualisation and writing – review & editing. **Alessandro Prete**: conceptualisation, funding acquisition, investigation, validation, visualisation and writing – review & editing. **Lorna C. Gilligan**: data curation, formal analysis, investigation, methodology, supervision, validation, visualisation and writing – review & editing. **Angela E. Taylor**: conceptualisation, funding acquisition, investigation, methodology, project administration, supervision, validation, visualisation, writing – original draft and writing – review & editing.

## Funding

This study received funding from the European Union's Horizon 2022 Research and Innovation Programme under Grant Agreement ID: 101095407, and through the Birmingham Biomedical Research Centre Grant ID: NIHR203326.

## Ethics Statement

All relevant study protocols complied with the Declaration of Helsinki and were approved by the local ethics committee, Science, Technology, Engineering and Mathematics Ethical Review Committee of the University of Birmingham, ERN_17‐0494.

## Conflicts of Interest

The authors declare no conflicts of interest.

## Supporting information




**Supporting File**: ansa70087‐sup‐0001‐SuppMat.docx.

## Data Availability

The data that support the findings of this study are available from the corresponding author upon reasonable request.
