# Supplementary material for: Comprehensive Quantitative Urinary Steroid Profiling of 29 Steroids Using Liquid Chromatography‐Tandem Mass Spectrometry
Source: Anal Sci Adv. 2026 Apr 30;7(1):e70087. doi: 10.1002/ansa.70087 (PMC13132653; doi:10.1002/ansa.70087)
Supplement: Supplementary file 1 — Supporting File: ansa70087‐sup‐0001‐SuppMat.docx. [file ANSA-7-e70087-s001.docx]

## **Supplementary Material**

## Title:

Comprehensive Quantitative Urinary Steroid Profiling of 29 Steroids Using Liquid Chromatography-Tandem Mass Spectrometry

## Authors

Joshua T. Bain (1), Fozia Shaheen (1), Alessandro Prete (1,2), Lorna C. Gilligan (1), Angela E. Taylor (1)

## Affiliation

1. Department of Metabolism and Systems Science, College of Medicine and Health, University of Birmingham, Birmingham, UK
2. National Institute for Health and Care Research (NIHR) Birmingham Biomedical Research Centre, United Kingdom.

## Corresponding Author

Dr. Angela E. Taylor
a.taylor.5@bham.ac.uk

## Supplementary Materials

## Contents

[Title: 1](#_Toc215563172)

[Authors 1](#_Toc215563173)

[Affiliation 1](#_Toc215563174)

[Corresponding Author 1](#_Toc215563175)

[Supplementary Materials 1](#_Toc215563176)

[Contents 2](#_Toc215563177)

[Table S1: Steroid nomenclature. 3](#_Toc215563178)

[Table S2: Concentrations of steroids in calibration series. 5](#_Toc215563179)

[Table S3: Percentage bias (average) comparing calibration series 6](#_Toc215563180)

[Table S4: Comparison of ion ratios (quantifier to qualifier) in calibration series. 7](#_Toc215563181)

[Table S5: Comparison of internal standard peak area deviation in calibration series to biological samples). 8](#_Toc215563182)

[Table S6: Urinary steroid average recovery, matrix effects, carry over, lower and upper limits of quantification accuracy and imprecision. 9](#_Toc215563183)

[Table S7: Steroid matrix effects in male, female and pooled urine samples. 11](#_Toc215563184)

[Table S8: Intra-assay (within-run) and inter-assay (between-run) imprecision of a male urine sample, a female urine sample and pooled urine. 12](#_Toc215563185)

[Table S9: Steroid stability data. 13](#_Toc215563186)

[Table S10: Median (interquartile ranges) for urine steroid excretion over 24 hours and during the day, and night and for male and female participants in µg/24hr (24hour). 14](#_Toc215563187)

[Table S11: Urinary steroid excretion expressed as median (interquartile range) for day, night and 24-hour collections from healthy males and females in nmol/L. 15](#_Toc215563188)

[Figure S1: Box and Whisker plot depicting urinary steroid excretion measured as nmol/L within a female population. 16](#_Toc215563189)

[Figure S2: Box and Whisker plot depicting urinary steroid excretion measured as nmol/L within a male population 17](#_Toc215563190)

### Table S1: Steroid nomenclature 29 steroids and 14 steroid internal standards including abbreviation, trivial and chemical name, functional class and suppliers.

| **Steroid abbreviation** | **Trivial name** | **Chemical name** | **Functional class** | **Supplier of standard** |
| --- | --- | --- | --- | --- |
| An | androsterone | 5α-androstan-3a-ol-17-one | Classic androgen | Steraloids |
| Et | etiocholanolone | 5β-androstan-3α-ol-17-one | Classic androgen | Merck Life Science |
| 11βOHAn | 11β-hydroxyetiocholanolone | 5α-androstane-3α,11β-diol-17-one | Androgen precursor/  11 oxygenated androgen | Steraloids |
| DHEA | dehydroepiandrosterone | 5β-adrostane-3α,11β-diol-17-one | Androgen precursor | Merck Life Science |
| 5PT | pregnenetriol | 5α-pregnene-3β,17α,20α-triol | Androgen precursor | Steraloids |
| 5PD | pregnenediol | 5α-pregnene-3β,20α-diol | Androgen precursor | Steraloids |
| THA + 5αTHA | tetrahydro-11-dehydrocorticosterone | 5-pregnane-3α,21-diol-11,20-dione | Mineralocorticoids | Steraloids |
| THB | tetrahydrocorticosterone | 5β-pregnane-3α,11β,21-triol-20-one | Mineralocorticoids | Steraloids |
| 5αTHB | 5α-tetrahydrocorticosterone | 5a-pregnane-3α,11β,21-triol-20-one | Mineralocorticoids | Steraloids |
| THAldo | tetrahydroaldosterone | 5-pregnan-3,11, 21-triol-20-one-18-al | Mineralocorticoids | Steraloids |
| THDOC | tetrahydro-11-deoxycorticosterone | 5β-pregnane-3α,21-diol-20-one | Mineralocorticoids | Steraloids |
| PD | pregnenediol | 5β-pregnene-3β,20α-diol | Glucocorticoid precursor | Merck Life Science |
| 17HP | 17α-hydroxypregnanolone | 5β-pregnane-3α,17α-diol-20-one | Glucocorticoid precursor | Steraloids |
| PT | pregnenetriol | 5β-pregnene-3β,17α,20α-triol | Glucocorticoid precursor | Steraloids |
| PTONE | pregnanetriolone | 5β-pregnane-3α,17α,20a-triol-11-one | Glucocorticoid precursor | Steraloids |
| THS | tetrahydro-11-deoxycortisol | 5β-pregnane-3α,17α,21-triol-20-one | Glucocorticoid precursor | Steraloids |
| F | cortisol | 4-pregnene-11β,17α,21-triol-3,20-dione | Glucocorticoid | Merck Life Science |
| THF | tetrahydrocortisol | 5β-pregnane-3α,11β,17α,21-tetrol-20-one | Glucocorticoid | Steraloids |
| 5αTHF | 5a-tetrahydrocortisol | 5a-pregnane-3α,11β,17α,21-tetrol-20-one | Glucocorticoid | Steraloids |
| β-cortol | β-cortol | 5β-pregnane-3α,11β,17α,20β,21-pentol | Glucocorticoid | Steraloids |
| α-cortol | α-cortol | 5β-pregnane-3α,11β,17α,20α,21-pentol | Glucocorticoid | Steraloids |
| 11βOHEt | 11β-hydroxyetiocholanolone | 5β-adrostane-3α,11β-diol-17-one | Glucocorticoid/  11 oxygenated androgen | Steraloids |
| 18OHF | 18-hydroxy-cortisol | 4-pregnene-11β,17α,18,21-tetraol-3,20-dione | Hybrid steroid | Steraloids |
| E | cortisone | 4-pregnene-17α,21-diol-3,11,20-trione | Glucocorticoid | Merck Life Science |
| THE | tetrahydrocortisone | 5β-pregnene-3α,17α,21-triol-11,20-dione | Glucocorticoid | Steraloids |
| α-cortolone | α-cortolone | 5β-pregnane-3a,17α,20α,21-tetrol-11-one | Glucocorticoid | Steraloids |
| β-cortolone | β-cortolone | 5β-pregnane-3α,17α,20β,21-tetrol-11-one | Glucocorticoid | Steraloids |
| 11OxoAn | 11-keto-androsterone | 5α-androstane-3α-ol-11,17-dione | Glucocorticoid/  11 oxygenated androgen | Steraloids |
| 11OxoEt | 11-keto-etiocholanolone | 5β-androstane-3α-ol-11,17-dione | Glucocorticoid/  11 oxygenated androgen | Steraloids |
| **Internal Standards** | | | | |
| **Steroid abbreviation** | **Trivial name** | **Chemical name** | **Functional class** | **Supplier of standard** |
| An-d4 | Androsterone-d4 | 5α-androstan-3a-ol-17-one-d4 | Classic androgen | Merck Life Science |
| Et-d5 | Etiocholanolone-d5 | 5β-androstan-3α-ol-17-one-d5 | Classic androgen | IsoSciences |
| DHEA-d6 | Dehydroepiandrosterone-d6 | 5β-adrostane-3α,11β-diol-17-one-d6 | Androgen precursor | Merck Life Science |
| THB-d5 | 5α-tetrahydrocorticosterone-d5 | 5a-pregnane-3α,11β,21-triol-20-one-d5 | Mineralocorticoids | IsoSciences |
| THAldo-d6 | Tetrahydroaldosterone-d6 | 5-pregnan-3,11, 21-triol-20-one-18-al-d6 | Mineralocorticoids | IsoSciences |
| PD-d5 | Pregnanediol-d5 | 5β-pregnene-3β,20α-diol-d5 | Glucocorticoid precursor | IsoSciences |
| PT-d5 | Pregnenetriol-d5 | 5β-pregnene-3β,17α,20α-triol-d5 | Glucocorticoid precursor | IsoSciences |
| THS-d5 | tetrahydro-11-deoxycortisol-d5 | 5β-pregnane-3α,17α,21-triol-20-one-d5 | Glucocorticoid precursor | Merck Life Science |
| F-d4 | Cortisol-d4 | 4-pregnene-11β,17α,21-triol-3,20-dione-d4 | Glucocorticoid | Merck Life Science |
| 18OHF-d4 | 18-hydroxy-cortisol-d4 | 4-pregnene-11β,17α,18,21-tetraol-3,20-dione-d4 | Hybrid steroid | IsoSciences |
| THF-d5 | Tetrahydrocortisol-d5 | 5β-pregnane-3α,11β,17α,21-tetrol-20-one-d5 | Glucocorticoid | IsoSciences |
| E-d7 | Cortisone-d7 | 4-pregnene-17α,21-diol-3,11,20-trione-d7 | Glucocorticoid | Merck Life Science |
| THE-d5 | Tetrahydrocortisone-d5 | 5β-pregnene-3α,17α,21-triol-11,20-dione-d5 | Glucocorticoid | IsoSciences |
| 11OxoEt-d5 | 11-keto-etiocholanolone-d5 | 5β-androstane-3α-ol-11,17-dione-d5 | Glucocorticoid/  11 oxygenated androgen | IsoSciences |

### Table S2: Concentrations of steroids in calibration series, group 1 steroids, 11OH β An, DHEA, 5PD, 5PT, THAs, THB, 5 α THB, THAldo, THDOC, PD, 17HP, PT, PTONE, THS, F, β-cortol, 11βOHEt,18OHF, E, α -cortolone, β-cortolone, 11OxoAn, 11OxoEt, group 2 steroids: An, Et, 5 α THF, THF and THE.

| **Calibration Point** | **Concentration Group 1**  **(ng/mL)** | **Concentration Group 2 (ng/mL)** |
| --- | --- | --- |
| C0 | 0 | 0 |
| C1 | 0.5 | 1 |
| C2 | 1 | 2 |
| C3 | 5 | 10 |
| C4 | 10 | 20 |
| C5 | 20 | 40 |
| C6 | 50 | 100 |
| C7 | 100 | 200 |
| C8 | 250 | 500 |
| C9 | 500 | 1000 |
| C10 | 1000 | 2000 |
| C11 | 2000 | 4000 |
| C12 | 3000 | 6000 |

### Table S3: Percentage bias (average) comparing calibration series prepared in spiked matrices (Surine, Sigmatrix Urine Diluent and 0.1%BSA in PBS) to theoretical concentrations.

| **Bias % (range)** | | | |
| --- | --- | --- | --- |
| **Analyte** | **SUrine** | **Sigmatrix Urine Diluent** | **0.1%BSA in PBS** |
| An | 2.1(-2.9-6.5) | 6.0(-0.9-11.5) | 7.2(-2.1-12.9) |
| Et | 4.1(-1.5-9.2) | 3.4(-1.9-9.5) | 2.2(-5.7-9.2) |
| 11βOHAn | 1.7(-7.5-7.3) | 0.5(-7.0-6.9) | -0.1(-8.3-6.3) |
| DHEA | 0.1(-9.3-11.7) | 1.9(-11.9-10.0) | 3.1(-8.5-12.3) |
| 5PD | 1.6(-9.1-15.8) | -0.5(-16.7-15.5) | 6.2(-10.1-16.2) |
| 5PT | -0.1(-1.9-4.3) | 1.3(-11.1-9.5) | -0.3(-10.3-13.4) |
| THAs | -0.2(-4.8-3.7) | 5.7(-3.8-15.0) | 4.0(-8.2-12.6) |
| THB | 2.0(-10.0-16.4) | 6.4(0.3-14.8) | 3.4(-6.0-11.1) |
| 5αTHB | 0.0(-10.1-8.2) | 2.4(-6.3-12.2) | 6.8(-10.9-17.9) |
| ThAldo | -2.7(-16.4-6.2) | 3.5(-14.8-18.4) | 5.6(-6.3-16.9) |
| THDOC | -4.3(-15.4-7.0) | -5.9(-17.5-14.0) | -1.5(-14.7-12.1) |
| PD | -4.3(-15.0-7.7) | -5.0(-13.1-6.3) | 5.9(0.0-16.7) |
| 17HP | -1.5(-10.9-17.6) | 2.7(-5.5-16.8) | 1.9(-4.3-14.8) |
| PT | 0.7(-2.9-9.8) | 1.3(-10.3-15.5) | 2.3(-0.5-6.3) |
| PTONE | -5.5(-16.1-4.7) | -4.0(-13.0-5.8) | -0.8(-16.3-15.5) |
| THS | 0.1(-5.2-7.2) | 5.6(-1.3-11.2) | 5.7(-1.2-13.0) |
| F | 3.1(-11.0-14.5) | 6.2(-0.6-14.3) | 2.9(-12.4-13.4) |
| THF | -11.8(-16.7--4.4) | -6.5(-17.9-1.8) | -0.8(-18.5-11.4) |
| 5αTHF | -9.6(-19.1-2.3) | -5.1(-13.3-4.7) | -3.0(-19.5-9.2) |
| β-cortol | -8.0(-19.6-5.6) | -6.4(-19.4-6.3) | -10.2(-18.8-4.6) |
| 11βOHEt | 4.1(-2.4-13.9) | 7.6(0.0-16.8) | 3.7(-16.1-16.4) |
| 18OHF | 6.2(-5.6-19.0) | 5.0(-12.5-17.3) | 7.4(-8.2-19.2) |
| E | 4.2(-3.1-9.2) | 6.0(-12.2-16.1) | 8.4(-7.1-14.0) |
| THE | -0.6(-4.4-7.8) | 5.8(0.8-13.5) | 1.4(-16.6-11.1) |
| α-cortolone | 5.4(-1.0-18.3) | 1.6(-16.0-13.3) | -5.4(-11.7--0.6) |
| β-cortolone | -2.4(-8.9-0.9) | -1.7(-6.6-3.1) | -6.7(-13.7--0.4) |
| 11oxoAn | 6.3(-6.4-16.3) | 0.8(-12.3-14.1) | 4.8(-4.2-13.8) |
| 11oxoEt | 4.7(-7.5-16.5) | 4.7(-11.4-15.9) | 3.2(-10.8-15.1) |

### Table S4: Comparison of average ion ratios (quantifier to qualifier) in calibration series (n=12) to biological samples (n=30).

| **Analyte** | **Average ion ratio in reference standards (range)** | **Average ion ratio in biological samples (range)** |
| --- | --- | --- |
| An | 1.7 (1.6-1.8) | 1.7 (1.7-1.8) |
| Et | 1.7 (1.6-1.8) | 1.6 (1.6-1.6) |
| 11βOHAn | 1.3 (1.0-3.8) | 1.3 (1.2-1.5) |
| DHEA | 0.8 (0.7-0.9) | 0.9 (0.7-1.1) |
| 5PD | 1.1 (0.0-2.3) | 1.0 (0.9-1.3) |
| 5PT | 2.6 (1.7-4.2) | 2.4 (2.1-2.9) |
| THAs | 5.1 (4.5-6.7) | 4.6 (4.1-5.0) |
| THB | 1.1 (0.7-1.3) | 1.1 (0.9-1.3) |
| 5αTHB | 1.0 (0.8-1.1) | 0.8 (0.8-0.9) |
| THAldo | 0.9 (0.8-1.0) | 0.9 (0.8-1.0) |
| THDOC | 1.0 (0.7-1.5) | 1.1 (0.8-1.2) |
| PD | 0.7 (0.4-1.3) | 0.7 (0.6-0.8) |
| 17HP | 6.0 (5.1-6.5) | 5.9 (5.1-6.4) |
| PT | 0.2 (0.2-0.2) | 0.2 (0.2-0.2) |
| PTONE | 1.6 (1.1-1.9) | 1.5 (1.3-1.7) |
| THS | 4.1 (2.6-4.5) | 3.8 (3.1-4.8) |
| F | 2.5 (2.4-2.7) | 2.3 (2.2-2.5) |
| THF | 2.3 (1.7-2.6) | 2.3 (2.1-2.6) |
| 5αTHF | 1.5 (1.5-1.7) | 1.4 (1.2-1.5) |
| α-cortol | n/a | 2.2 (2.0-2.3) |
| β-cortol | 0.3 (0.3-0.3) | 0.3 (0.3-0.3) |
| 11βOHEt | 0.9 (0.8-1.0) | 0.9 (0.7-1.0) |
| 18OHF | 2.2 (2.1-2.2) | 2.2 (2.1-2.3) |
| E | 3.4 (3.3-3.4) | 3.3 (3.2-3.3) |
| THE | 2.0 (1.9-2.1) | 2.0 (1.9-2.0) |
| α-cortolone | 1.3 (1.2-1.4) | 1.3 (1.2-1.4) |
| β-cortolone | 1.3 (1.2-1.3) | 1.5 (1.3-1.5) |
| 11oxoAn | 1.7 (1.3-1.9) | 1.8 (1.4-2.1) |
| 11oxoEt | 1.1 (0.8-1.4) | 1.2 (1.1-1.4) |

### Table S5: Comparison of internal standard peak area deviation in calibration series (n=12) to biological samples (n=30) percent variation (% deviation from average peak area in calibration series).

| **Internal standard** | **Average deviation %**  **(range)** |
| --- | --- |
| An-d4 | 3.0 (-7.7-8.8) |
| Et-d5 | 1.1 (-11.2-5.7) |
| DHEA-d6 | -5.3 (-14.4-3.4) |
| THB-d5 | -17.5 (-19.8--12.9) |
| THAldo-d6 | 3.6 (-10.7-16.5) |
| PD-d5 | 1.2 (-15.4-15.8) |
| PT-d5 | 4.3 (-10.0-9.5) |
| THS-d5 | -10.0 (-19.6--4.1) |
| F-d4 | 2.4 (-8.2-11.8) |
| 18OHF-d4 | -3.7 (-15.8-4.6) |
| E-d7 | 1.7 (-12.6-13.3) |
| THE-d5 | 1.2 (-9.1-8.4) |
| OxoEt-d5 | -0.4 (-9.5-12.8) |
| THF-d5 | -6.0 (-19.4-1.9) |

### Table S6: Urinary steroid average recovery, matrix effects, carry over, lower and upper limits of quantification accuracy and imprecision. Recovery and matrix effects data compiled from male, female and pooled urine samples. Carry over in a blank sample following the top calibrant. The lower limit of quantification (LLOQ) was defined as the lowest quantifiable concentration with accuracy (bias within ±20%) and imprecision (CV <20%). The upper limit of quantification (ULOQ) was defined as the highest concentration with accuracy (bias within ±15%) and imprecision (CV <15%), both converted to 24-hour concentrations using a typical 24hr urine volume of 1400mL. Accuracy and imprecision were determined at three concentrations low, medium and high (20, 300 and 800ng/mL) (40, 600 and 1600ng/mL for An, Et, 5αTHF, THF and THE) spiked into surrogate matrix. Due to lack of standard for α-cortol this was not assessed for accuracy or precision.

| **Steroid** | **Avg Recovery (%)** | **Avg**  **Matrix Effects (%)** | **Carry over (%)** | **LLOQ ng/mL (µg/24hr)** | **ULOQ µg/mL (mg/24hr)** | **Accuracy Bias (%)** | | | | | | **Imprecision CV (%)** | | | | |
| --- | --- | --- | --- | --- | --- | --- | --- | --- | --- | --- | --- | --- | --- | --- | --- | --- |
|  |  |  |  |  |  | **LLOQ** | **Low** | **Med** | **High** | **ULOQ** | **LLOQ** | | **Low** | **Med** | **High** | **ULOQ** |
| An | 85 | 5.0 | 0.3 | 10 (14) | 6 (8.4) | 11.8 | -7.0 | -1.8 | 6.1 | -14.0 | 11.7 | | 11.8 | 7.1 | 4.4 | 2.2 |
| Et | 79 | 7.9 | 0.3 | 10 (14) | 6 (8.4) | -18.7 | 8.2 | -11.8 | 2.6 | 10.8 | 9.1 | | 10.2 | 7.2 | 4.9 | 2.1 |
| 11βOHAn | 87 | 3.0 | 0.4 | 5 (7) | 3 (4.2) | -12.0 | 9.2 | -13.7 | -8.6 | -3.8 | 18.6 | | 8.9 | 6.1 | 4.5 | 1.6 |
| DHEA | 86 | 4.9 | 0.3 | 5 (7) | 3 (4.2) | -9.6 | 7.0 | -7.8 | -4.8 | -8.6 | 9.2 | | 12.4 | 7.4 | 5.6 | 1.2 |
| 5PT | 86 | 7.2 | 0.2 | 10 (14) | 3 (4.2) | 11.9 | 4.5 | -2.1 | 4.6 | -5.9 | 4.0 | | 10.2 | 5.7 | 5.3 | 1.5 |
| 5PD | 77 | 8.9 | 0.1 | 20 (28) | 3 (4.2) | 19.1 | 19.1 | -18.8 | -1.8 | -6.2 | 11.1 | | 11.1 | 12.4 | 8.0 | 3.0 |
| THAs | 86 | 2.3 | 0.1 | 5 (7) | 3 (4.2) | 12.8 | 10.7 | -1.8 | -0.4 | -13.7 | 6.4 | | 12.6 | 7.1 | 4.7 | 2.0 |
| THB | 89 | 5.0 | 0.2 | 5 (7) | 3 (4.2) | 7.7 | 14.8 | -1.8 | -1.7 | -8.7 | 5.8 | | 6.4 | 6.5 | 4.8 | 1.8 |
| 5αTHB | 87 | 5.4 | 0.4 | 2 (2.8) | 3 (4.2) | 3.7 | 8.7 | 8.9 | -2.8 | -9.9 | 12.6 | | 11.3 | 6.9 | 4.6 | 1.8 |
| THAldo | 92 | 3.4 | 0.2 | 5 (7) | 3 (4.2) | -0.3 | 5.7 | 6.2 | 4.9 | -13.1 | 5.7 | | 12.7 | 5.1 | 3.7 | 1.3 |
| THDOC | 92 | 3.6 | 0.5 | 10 (14) | 3 (4.2) | -4.2 | -5.0 | -4.1 | 7.8 | 14.5 | 12.0 | | 7.8 | 7.4 | 8.1 | 2.5 |
| PD | 92 | 4.3 | 1.9 | 5 (7) | 3 (4.2) | 8.3 | 3.3 | 6.2 | -2.4 | -7.1 | 11.7 | | 10.1 | 9.4 | 6.9 | 9.5 |
| 17HP | 82 | 0.2 | 0.4 | 5 (7) | 3 (4.2) | 19.9 | 12.7 | -4.9 | 14.8 | -7.7 | 6.6 | | 12.1 | 7.2 | 6.2 | 1.4 |
| PT | 78 | 6.0 | 0.2 | 5 (7) | 3 (4.2) | -12.6 | -2.4 | 0.1 | 4.1 | -10.4 | 10.4 | | 13.0 | 7.1 | 4.7 | 2.5 |
| PTONE | 92 | 6.2 | 0.1 | 5 (7) | 3 (4.2) | -6.2 | 13.1 | -12.4 | 1.7 | -2.5 | 9.9 | | 11.8 | 6.1 | 4.5 | 1.6 |
| THS | 86 | 7.6 | 0.2 | 5 (7) | 3 (4.2) | 4.3 | 11.4 | 2.3 | 1.7 | -12.0 | 17.1 | | 11.2 | 7.5 | 5.4 | 1.2 |
| F | 87 | 8.0 | 0.5 | 5 (7) | 3 (4.2) | 3.3 | 14.5 | 5.8 | 2.4 | -10.0 | 6.5 | | 11.6 | 6.7 | 3.5 | 1.3 |
| THF | 91 | 2.4 | 0.3 | 10 (14) | 6 (8.4) | 13.7 | 2.2 | 6.4 | 1.8 | -12.4 | 5.8 | | 13.7 | 7.2 | 3.8 | 2.0 |
| 5αTHF | 91 | 3.0 | 0.2 | 10 (14) | 6 (8.4) | 19.1 | 9.0 | 6.8 | -0.1 | -12.3 | 6.5 | | 10.1 | 6.1 | 4.4 | 1.9 |
| β-cortol | 103 | 7.9 | 0.1 | 5 (7) | 3 (4.2) | 3.8 | 12.7 | -14.9 | 7.2 | -6.3 | 5.6 | | 12.6 | 6.6 | 4.5 | 2.8 |
| 11βOHEt | 76 | 5.8 | 0.2 | 5 (7) | 3 (4.2) | -13.8 | 10.3 | -9.7 | -4.9 | -9.2 | 10.0 | | 14.1 | 7.1 | 4.0 | 1.3 |
| 18OHF | 92 | 7.4 | 0.4 | 2 (2.8) | 3 (4.2) | -7.7 | 11.5 | 3.1 | 3.6 | -11.5 | 12.3 | | 11.5 | 6.7 | 4.3 | 0.9 |
| E | 88 | 6.2 | 0.4 | 5 (7) | 3 (4.2) | 12.7 | 10.0 | 3.8 | -4.3 | -12.2 | 6.6 | | 12.1 | 6.5 | 3.4 | 1.4 |
| THE | 88 | 3.6 | 0.3 | 10 (14) | 6 (8.4) | -8.0 | 5.6 | -7.9 | -2.9 | -7.4 | 5.5 | | 11.3 | 7.1 | 4.1 | 0.9 |
| α-cortolone | 98 | 2.3 | 0.2 | 5 (7) | 3 (4.2) | -5.0 | 12.0 | -3.6 | -7.3 | -3.7 | 16.1 | | 11.7 | 8.0 | 4.5 | 2.7 |
| β-cortolone | 88 | 7.9 | 0.2 | 5 (7) | 3 (4.2) | -6.6 | 14.9 | -6.0 | 3.5 | -6.7 | 6.6 | | 6.6 | 7.5 | 4.6 | 0.9 |
| 11OxoAn | 87 | 3.0 | 0.2 | 5 (7) | 3 (4.2) | -8.5 | 6.2 | 1.6 | 4.7 | -14.1 | 12.0 | | 10.7 | 6.5 | 4.6 | 2.4 |
| 11OxoEt | 86 | 2.4 | 0.2 | 2 (2.8) | 3 (4.2) | -8.7 | 11.7 | 7.2 | 4.7 | -14.3 | 17.7 | | 12.1 | 5.0 | 3.8 | 2.5 |

### Table S7: Steroid matrix effects in male, female and pooled urine samples at three concentrations; 50ng/m (low), 100ng/mL (medium) and 200ng/mL (high).

| **Analyte** | **Matrix Effects (%)** | | | | | | | | |
| --- | --- | --- | --- | --- | --- | --- | --- | --- | --- |
|  | **Male** | | | **Female** | | | **Pooled** | | |
|  | **Low** | **Medium** | **High** | **Low** | **Medium** | **High** | **Low** | **Medium** | **High** |
| An | -1.4 | 14.7 | 13.3 | -8.8 | -3.1 | 8.5 | -4.8 | 13.7 | 12.6 |
| Et | 9.0 | 10.1 | 9.6 | 5.1 | 1.9 | 13.7 | -5.4 | 13.9 | 13.5 |
| 11βOHAn | -0.7 | -10.4 | -7.1 | 7.7 | 8.1 | -2.0 | 10.5 | 12.6 | 8.1 |
| DHEA | 1.7 | 9.4 | 7.1 | -15.0 | 8.7 | 2.9 | 13.9 | 3.1 | 12.4 |
| 5PD | 5.0 | 10.7 | 11.1 | 0.2 | 3.8 | 13.1 | 1.5 | 6.9 | 11.9 |
| 5PT | 11.8 | 14.7 | 12.7 | 1.7 | 6.0 | 7.8 | 7.0 | 6.9 | 11.5 |
| THAs | 3.9 | 8.8 | 3.9 | 8.9 | 4.2 | -3.4 | -7.4 | 0.1 | 2.0 |
| THB | 9.5 | 6.3 | 9.5 | -0.6 | -3.4 | -0.5 | 9.0 | 7.0 | 8.2 |
| 5αTHB | 11.8 | 10.0 | 2.2 | 1.0 | -4.0 | -2.5 | 14.1 | 12.0 | 3.8 |
| ThAldo | 13.3 | 14.9 | 7.7 | -13.9 | -14.4 | -11.3 | 11.3 | 10.7 | 12.3 |
| THDOC | 9.5 | 3.0 | 12.3 | -1.0 | 8.2 | 4.9 | -6.9 | 2.3 | 0.0 |
| PD | 6.8 | 14.1 | 9.0 | -2.9 | -10.2 | 6.6 | 6.4 | 0.3 | 8.4 |
| 17HP | -1.7 | 2.2 | 5.1 | -8.3 | -7.7 | 8.0 | -13.8 | 3.2 | 14.6 |
| PT | 3.1 | 12.9 | 14.6 | -0.9 | 12.5 | 12.4 | -6.0 | 0.3 | 5.3 |
| PTONE | 3.2 | 8.2 | -1.9 | -4.1 | 5.3 | 11.0 | 11.7 | 9.1 | 13.8 |
| THS | 7.6 | 14.8 | 8.9 | 9.4 | 1.4 | 5.1 | 0.9 | 13.3 | 7.0 |
| F | 7.2 | 5.4 | 6.7 | -0.7 | 6.9 | 14.6 | 11.1 | 10.0 | 11.0 |
| THF | -12.6 | 13.8 | 6.8 | -11.8 | -1.6 | 10.1 | 2.5 | 5.2 | 9.0 |
| 5αTHF | 5.6 | 7.4 | 11.1 | 7.1 | -3.1 | -8.4 | 1.0 | 5.0 | 1.2 |
| β-cortol | 11.5 | 4.8 | 11.1 | 12.9 | 11.7 | 13.9 | -3.7 | -4.6 | 13.8 |
| 11βOHEt | 5.0 | 3.4 | 8.4 | -8.6 | 9.4 | 10.6 | 3.3 | 5.7 | 14.6 |
| 18OHF | 4.5 | 12.5 | 13.2 | -5.7 | 8.1 | 11.7 | 2.5 | 7.8 | 12.1 |
| E | 14.6 | 12.1 | 10.6 | 1.4 | 6.9 | 12.1 | 6.1 | 12.7 | 14.4 |
| THE | -1.4 | 2.5 | 9.4 | 13.7 | 8.6 | 12.2 | -12.2 | 12.5 | 13.5 |
| α-cortolone | 0.6 | 3.0 | 11.8 | 10.1 | 12.3 | 8.5 | 3.9 | 7.8 | 13.5 |
| β-cortolone | 2.3 | 7.0 | 12.3 | -6.7 | 9.8 | 9.0 | 10.8 | 12.6 | 14.6 |
| 11oxoAn | -7.6 | -1.5 | -8.0 | -5.0 | 0.3 | -14.4 | -10.5 | -9.0 | 1.5 |
| 11oxoEt | 1.0 | 6.5 | -7.1 | -13.2 | 8.6 | -14.1 | -8.2 | -12.9 | 3.9 |

### Table S8: Intra-assay (within-run) and inter-assay (between-run) imprecision of a male urine sample, a female urine sample and pooled urine sample for all analytes.

| **Analyte** | **Male Urine** | | | **Female Urine** | | | **Pooled Urine** | | |
| --- | --- | --- | --- | --- | --- | --- | --- | --- | --- |
|  | **Mean (µg/24hr)** | **Intra-assay CV (%)** | **Inter-assay CV (%)** | **Mean (µg/24hr)** | **Intra-assay CV (%)** | **Inter-assay CV (%)** | **Mean (µg/24hr)** | **Intra-assay CV (%)** | **Inter-assay CV (%)** |
| An | 2088 | 2.0 | 9.8 | 2879 | 3.3 | 5.6 | 1515 | 2.6 | 2.9 |
| Et | 1880 | 2.3 | 9.4 | 2937 | 3.5 | 3.1 | 1065 | 2.9 | 3.3 |
| 11βOHAn | 623 | 1.2 | 2.1 | 683 | 6.1 | 14.6 | 405 | 2.6 | 8.6 |
| DHEA | 222 | 3.2 | 14.2 | 61 | 9.2 | 10.3 | 57 | 4.6 | 4.1 |
| 5PT | 138 | 4.6 | 5.7 | 121 | 4.1 | 12.8 | 105 | 5.2 | 8.5 |
| 5PD | 34 | 5.5 | 14.4 | 90 | 11.5 | 10.8 | 26 | 8.6 | 10.4 |
| THAs | 118 | 1.9 | 14.6 | 152 | 4.8 | 14.5 | 85 | 3.1 | 7.4 |
| THB | 100 | 3.1 | 8.1 | 136 | 12.1 | 10.0 | 45 | 5.1 | 14.6 |
| 5αTHB | 241 | 2.2 | 4.1 | 335 | 5.1 | 6.8 | 211 | 3.3 | 10.5 |
| THAldo | 22 | 5.1 | 14.9 | 55 | 3.3 | 12.8 | 17 | 2.8 | 8.4 |
| THDOC | 218 | 0.8 | 14.3 | 1107 | 4.7 | 14.5 | 248 | 3.2 | 8.8 |
| PD | 220 | 4.1 | 13.2 | 485 | 5.9 | 13.5 | 108 | 6.0 | 8.6 |
| 17HP | 114 | 2.3 | 12.3 | 107 | 4.5 | 4.8 | 89 | 3.5 | 3.4 |
| PT | 557 | 1.6 | 11.2 | 496 | 4.2 | 5.5 | 342 | 2.3 | 2.8 |
| PTONE | 4 | 9.6 | 12.1 | 8 | 10.0 | 9.6 | 8 | 12.6 | 12.9 |
| THS | 30 | 5.7 | 7.8 | 39 | 4.8 | 9.1 | 35 | 4.8 | 14.5 |
| F | 72 | 1.5 | 4.1 | 91 | 3.5 | 4.5 | 40 | 3.1 | 4.4 |
| THF | 1186 | 2.2 | 4.8 | 1630 | 3.7 | 4.4 | 853 | 3.2 | 7.6 |
| 5αTHF | 876 | 1.5 | 11.8 | 1137 | 2.3 | 13.0 | 844 | 2.7 | 14.8 |
| α-cortol | 63 | 3.2 | 13.2 | 124 | 4.0 | 6.9 | 37 | 6.7 | 6.9 |
| β-cortol | 236 | 1.6 | 5.3 | 348 | 4.0 | 7.1 | 350 | 4.7 | 8.3 |
| 11βOHEt | 258 | 6.1 | 14.4 | 207 | 8.9 | 8.6 | 77 | 9.7 | 12.6 |
| 18OHF | 116 | 1.2 | 4.3 | 63 | 3.4 | 3.5 | 45 | 3.6 | 8.0 |
| E | 114 | 2.1 | 9.8 | 178 | 3.0 | 5.8 | 62 | 2.6 | 3.5 |
| THE | 2395 | 2.2 | 3.8 | 2299 | 3.0 | 4.2 | 1693 | 2.9 | 7.0 |
| α-cortolone | 1004 | 1.4 | 3.7 | 1257 | 3.5 | 12.6 | 580 | 3.7 | 13.9 |
| β-cortolone | 291 | 2.1 | 5.7 | 244 | 3.8 | 7.6 | 269 | 3.0 | 4.8 |
| 11OxoAn | 47 | 1.9 | 6.5 | 44 | 8.5 | 9.1 | 23 | 8.5 | 7.4 |
| 11OxoEt | 308 | 2.3 | 6.6 | 239 | 3.3 | 3.9 | 135 | 3.0 | 6.1 |

### Table S9: Steroid stability data, based on pooled 24-hour urine. Imprecision CV (%) after 3 freeze/thaw cycles, after 2 months post preparation and when monitored long term- over 18-month storage at -20°C.

|  | **Imprecision CV (%)** | | |
| --- | --- | --- | --- |
| **Analyte** | **Freeze Thaw Cycle** | **Post-Preparative** | **Long term Biological Storage** |
| An | 2.6 | 11.5 | 11.5 |
| Et | 2.6 | 4.5 | 14.0 |
| 11βOHAn | 4.5 | 5.5 | 12.4 |
| DHEA | 10.7 | 13.3 | 13.5 |
| 5PT | 4.4 | 13.0 | 13.1 |
| 5PD | 8.3 | 13.2 | 13.2 |
| THAs | 10.7 | 11.8 | 12.3 |
| THB | 8.9 | 11.1 | 14.5 |
| 5αTHB | 10.2 | 8.5 | 14.6 |
| THAldo | 11.1 | 12.4 | 12.8 |
| THDOC | 11.8 | 13.1 | 11.7 |
| PD | 3.9 | 11.3 | 14.0 |
| 17HP | 3.1 | 3.2 | 14.0 |
| PT | 3.9 | 4.2 | 11.7 |
| PTONE | 7.5 | 14.8 | 14.6 |
| THS | 4.1 | 5.2 | 10.5 |
| F | 5.2 | 5.6 | 13.4 |
| THF | 2.9 | 6.9 | 14.6 |
| 5αTHF | 3.1 | 6.4 | 14.8 |
| α-cortol | 5.5 | 7.1 | 14.1 |
| β-cortol | 3.2 | 5.7 | 14.1 |
| 11βOHEt | 7.8 | 8.5 | 12.9 |
| 18OHF | 6.2 | 11.2 | 13.8 |
| E | 3.7 | 4.7 | 12.4 |
| THE | 2.3 | 4.4 | 9.6 |
| α-cortolone | 1.9 | 5.9 | 13.6 |
| β-cortolone | 2.7 | 5.3 | 11.5 |
| 11OxoAn | 5.4 | 9.4 | 14.6 |
| 11OxoEt | 5.8 | 9.7 | 14.3 |

###

### Table S10: Median (interquartile ranges) for urine steroid excretion over 24 hours and during the day, and night and for male and female participants in µg/24hr (24hour) and µg/sample (day or night).

| **Analyte** | **Female Median (IQR)** | | | **Male Median (IQR)** | | |
| --- | --- | --- | --- | --- | --- | --- |
|  | **24hr (µg/24hr)** | **Day (µg/sample)** | **Night (µg/sample)** | **24hr (µg/24hr)** | **Day (µg/sample)** | **Night (µg/sample)** |
| An | 1279(597-1742) | 799(402-1399) | 381(153-633) | 3819(2433-4860) | 2533(1663-3385) | 1049(764-1588) |
| Et | 1351(685-2020) | 887(524-1194) | 381(221-827) | 2646(1966-4353) | 1664(1327-3039) | 999(583-1411) |
| 11βOHAn | 655(509-854) | 451(365-649) | 185(117-321) | 1244(1024-1458) | 876(707-1129) | 329(223-442) |
| DHEA | 42(26-54) | 25(19-42) | 12(7-19) | 75(40-161) | 51(29-135) | 19(12-34) |
| 5PT | 146(69-252) | 100(55-179) | 46(20-79) | 258(174-385) | 171(125-288) | 78(50-109) |
| 5PD | 48(13-88) | 29(10-53) | 11(6-30) | 89(31-134) | 56(8-82) | 30(18-39) |
| THAs | 137(71-209) | 88(49-139) | 39(27-61) | 200(146-272) | 129(108-205) | 50(36-69) |
| THB | 113(60-243) | 65(38-137) | 41(19-70) | 151(118-208) | 100(73-170) | 42(34-64) |
| 5αTHB | 223(171-316) | 163(140-200) | 51(38-89) | 465(351-586) | 381(249-463) | 103(84-157) |
| THAldo | 43(33-66) | 32(24-46) | 10(8-20) | 52(30-66) | 34(23-50) | 13(7-20) |
| THDOC | 80(45-144) | 48(36-96) | 27(14-50) | 213(149-315) | 149(108-191) | 68(48-102) |
| PD | 253(127-504) | 135(71-369) | 80(32-185) | 262(209-423) | 186(138-279) | 93(61-151) |
| 17HP | 84(47-123) | 53(25-87) | 27(8-55) | 197(134-323) | 147(90-225) | 73(39-106) |
| PT | 416(261-597) | 261(171-368) | 160(60-249) | 973(702-1382) | 668(459-856) | 310(222-474) |
| PTONE | 7(4-15) | 5(2-11) | 2(1-4) | 15(9-40) | 10(6-32) | 5(2-10) |
| THS | 65(39-90) | 40(26-58) | 23(12-31) | 68(47-91) | 50(34-69) | 17(13-26) |
| F | 68(53-96) | 46(34-56) | 18(14-26) | 84(72-116) | 62(48-76) | 19(16-36) |
| THF | 1646(1078-2075) | 1194(737-1378) | 367(265-723) | 2474(1861-2884) | 1962(1304-2481) | 555(449-725) |
| 5αTHF | 545(392-827) | 413(308-685) | 111(82-198) | 1718(1198-2025) | 1353(940-1784) | 322(220-427) |
| α-cortol | 64(45-122) | 45(33-89) | 19(11-35) | 98(80-141) | 73(55-112) | 23(17-37) |
| β-cortol | 334(301-504) | 251(178-383) | 119(74-146) | 717(504-978) | 540(367-717) | 177(128-287) |
| 11βOHEt | 274(205-619) | 169(124-407) | 123(75-201) | 359(76-578) | 220(30-363) | 88(46-220) |
| 18OHF | 65(50-84) | 44(30-58) | 21(15-32) | 102(89-130) | 64(58-98) | 31(23-42) |
| E | 106(73-133) | 66(45-94) | 30(22-51) | 125(105-155) | 89(74-116) | 30(24-53) |
| THE | 2260(1469-3048) | 1571(980-2024) | 575(409-1022) | 4161(3492-5104) | 2973(2502-3893) | 985(740-1238) |
| α-cortolone | 954(685-1125) | 600(515-878) | 217(144-355) | 1464(1184-1787) | 1110(855-1501) | 301(218-485) |
| β-cortolone | 405(241-456) | 277(154-352) | 81(64-154) | 668(532-830) | 533(385-617) | 158(109-230) |
| 11OxoAn | 52(40-73) | 35(29-41) | 18(10-20) | 75(63-98) | 57(47-68) | 22(17-25) |
| 11OxoEt | 322(267-650) | 229(184-414) | 110(64-203) | 444(208-920) | 293(134-501) | 151(78-275) |

### Table S11: Urinary steroid excretion expressed as median (interquartile range) for day, night and 24-hour collections from healthy males and females converted to nmol/L.

| **Analyte** | **Female Median (IQR)** | | | **Male Median (IQR)** | | |
| --- | --- | --- | --- | --- | --- | --- |
|  | **24hr (nmol/L)** | **Day (nmol/L)** | **Night (nmol/L)** | **24hr (nmol/L)** | **Day (nmol/L)** | **Night (nmol/L)** |
| An | 2237(945-4261) | 2179(1008-3575) | 2360(1300-5030) | 6588(3604-10825) | 6878(3430-9110) | 8359(5613-13707) |
| Et | 2220(1535-4507) | 2023(1421-3447) | 2765(1409-6898) | 4980(3387-7141) | 5021(3123-6662) | 7111(4153-9768) |
| 11βOHAn | 1171(928-1437) | 1274(852-1846) | 1278(870-2342) | 2126(1329-2750) | 2241(1155-2762) | 2373(1256-3661) |
| DHEA | 75(31-128) | 87(42-132) | 81(27-160) | 136(77-263) | 133(80-250) | 142(89-308) |
| 5PT | 286(108-418) | 270(96-437) | 295(101-480) | 408(287-619) | 434(268-637) | 451(285-873) |
| 5PD | 85(21-165) | 81(26-108) | 66(34-249) | 150(38-252) | 134(12-228) | 207(101-280) |
| THAs | 202(121-360) | 186(120-353) | 223(124-321) | 331(156-487) | 320(151-489) | 333(189-504) |
| THB | 161(92-442) | 148(88-402) | 189(115-546) | 248(182-357) | 273(155-338) | 310(205-471) |
| 5αTHB | 314(237-561) | 359(253-666) | 367(203-575) | 674(409-1025) | 808(455-1015) | 743(446-1046) |
| THAldo | 66(49-94) | 72(53-102) | 79(43-115) | 76(40-101) | 80(33-111) | 83(45-120) |
| THDOC | 127(61-254) | 130(64-216) | 139(78-293) | 324(208-536) | 322(174-539) | 442(301-656) |
| PD | 493(165-1108) | 359(156-1085) | 636(179-1715) | 462(281-692) | 400(238-668) | 639(391-861) |
| 17HP | 106(69-256) | 96(63-213) | 172(63-257) | 355(184-805) | 331(183-700) | 427(265-864) |
| PT | 712(398-1031) | 603(382-893) | 822(509-1477) | 1521(927-2071) | 1291(904-2000) | 2194(1244-3459) |
| PTONE | 12(6-25) | 13(4-29) | 14(4-27) | 20(12-56) | 20(10-67) | 21(15-74) |
| THS | 98(53-133) | 100(50-134) | 101(61-211) | 100(72-154) | 90(81-150) | 104(76-158) |
| F | 102(68-123) | 99(72-163) | 121(66-203) | 119(82-196) | 121(77-222) | 150(98-212) |
| THF | 2167(1272-3060) | 2403(1294-3734) | 2462(1321-3872) | 3316(2453-5019) | 3846(2362-5055) | 3556(2523-5061) |
| 5αTHF | 829(559-1318) | 1112(589-1402) | 736(426-1222) | 2416(1495-3300) | 2391(1590-3124) | 2240(1159-2886) |
| α-cortol | 97(58-158) | 113(55-197) | 114(60-233) | 146(98-235) | 156(93-217) | 200(80-319) |
| β-cortol | 502(416-773) | 552(348-1291) | 562(400-1212) | 1419(537-1887) | 1605(610-1722) | 1167(718-2402) |
| 11βOHEt | 554(314-1033) | 690(249-1208) | 921(441-1433) | 552(149-963) | 387(83-1128) | 943(279-1673) |
| 18OHF | 99(81-142) | 96(66-189) | 187(64-247) | 181(121-269) | 172(105-282) | 234(167-367) |
| E | 142(112-175) | 141(112-268) | 173(115-305) | 192(131-257) | 181(130-262) | 244(147-299) |
| THE | 2869(2208-4561) | 3138(2296-5331) | 3765(1991-5057) | 5832(4728-8351) | 6354(4564-8426) | 5949(3532-8204) |
| α-cortolone | 1235(954-1940) | 1453(891-2095) | 1274(875-2466) | 1957(1227-3250) | 2088(1396-3173) | 2238(1043-3760) |
| β-cortolone | 536(335-736) | 547(330-800) | 492(369-775) | 1081(634-1389) | 1134(632-1348) | 918(574-1808) |
| 11OxoAn | 90(59-124) | 100(66-146) | 128(65-187) | 146(97-168) | 143(92-186) | 157(117-212) |
| 11OxoEt | 547(406-785) | 613(361-836) | 656(468-856) | 551(349-932) | 563(315-755) | 839(413-1551) |


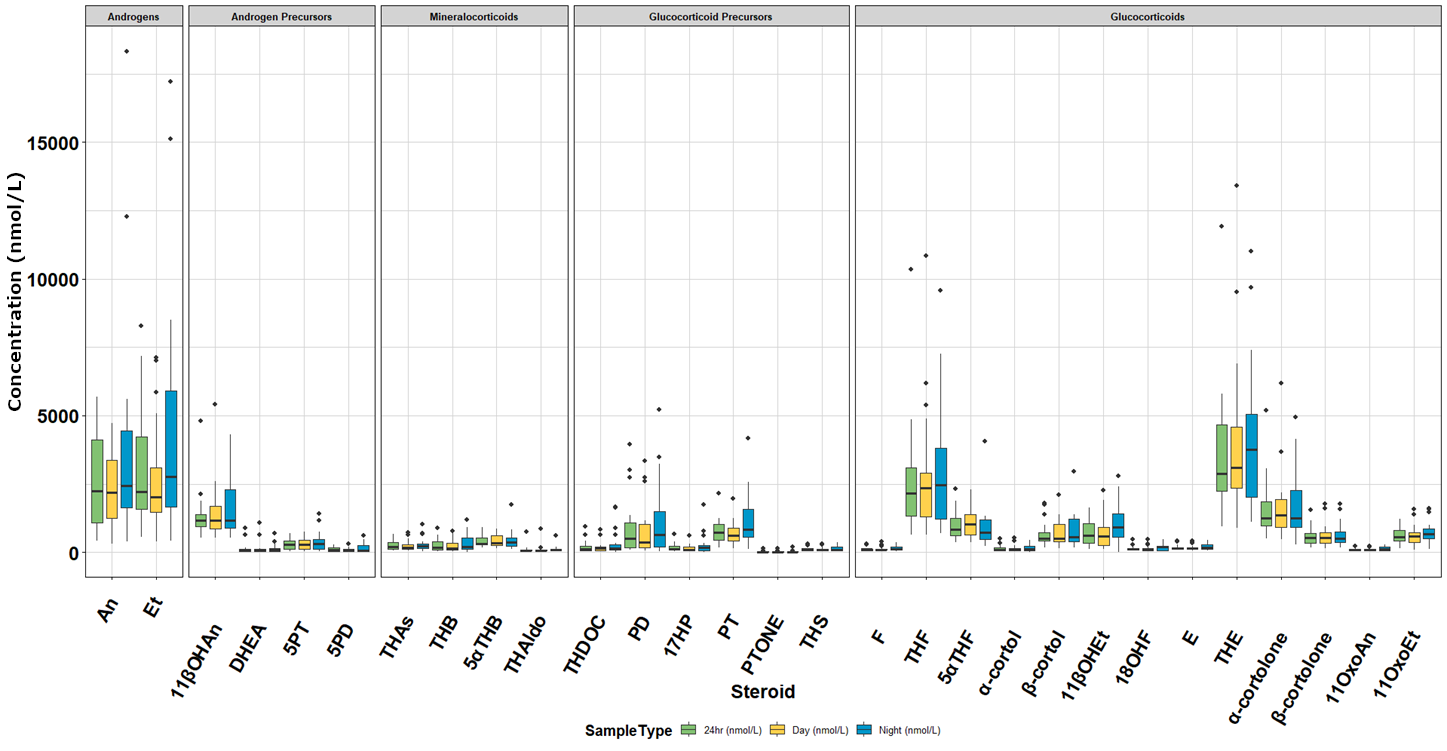


### Figure S1: Box and Whisker plot depicting urinary steroid excretion measured as nmol/L within a female population (n=20) 24hr (green), day (yellow), and night (blue).


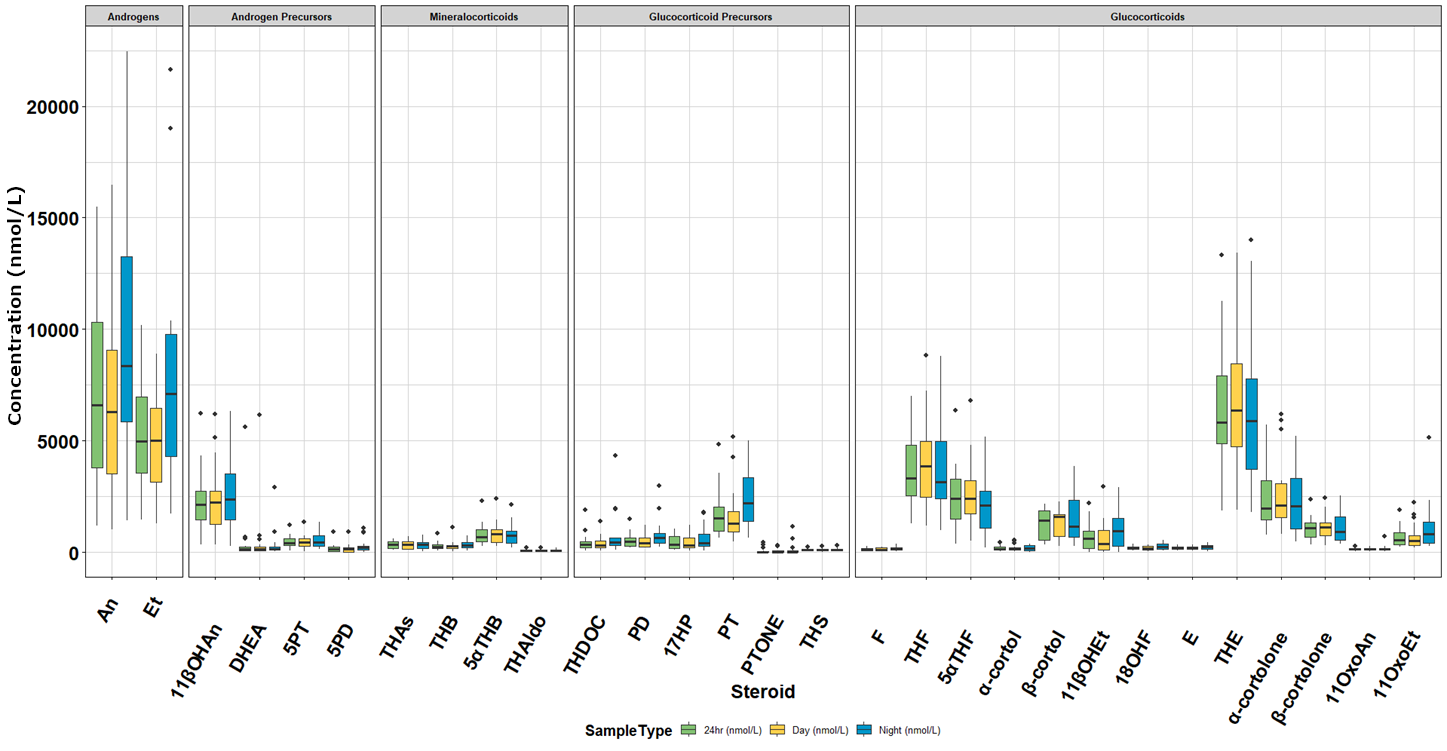


### Figure S2: Box and Whisker plot depicting urinary steroid excretion measured as nmol/L within a male population (n=20) 24hr (green), day (yellow), and night (blue).
